# Clinical efficacy of a Chinese herbal gel plaster combined with manipulation for lumbar disc herniation: a prospective, randomized, double-blind, placebo-controlled trial

**DOI:** 10.3389/fphar.2026.1708694

**Published:** 2026-03-19

**Authors:** Tianhao Wan, Gezhi Zhang, Haibao Wen, Lifeng Zhuang, Haotian Yang, Di Xia, Chao Su, Jinqiao Zou, Fajie Li, Jinyu Gu, Qing Zhang

**Affiliations:** 1 Department of Spine II, Wangjing Hospital, China Academy of Chinese Medical Sciences, Beijing, China; 2 Standardization Research Office, Institute of Basic Research in Clinical Medicine, China Academy of Chinese Medical Sciences, Beijing, China; 3 Department of Trauma Orthopedics, Xiyuan Hospital of China Academy of Chinese Medical Sciences, Beijing, China

**Keywords:** Haitongpi formula gel plaster, low back pain, lumbar disc herniation, randomized controlled trial, analgesic effect

## Abstract

**Background:**

Haitongpi Formula, derived from the Traditional Chinese Medicine (TCM) classic Yizong Jinjian, has shown efficacy for low back pain, fractures, and osteoarthritis. Haitongpi Formula Gel Paste (HTPGP) is a modern dosage form of this formula, but clinical evidence on its effectiveness and safety remains limited. This study was designed to evaluate the clinical efficacy and safety of HTPGP combined with manipulation in relieving chronic pain caused by lumbar disc herniation (LDH).

**Methods:**

Adults aged 40–60 years with LDH were randomly assigned to receive Qing dynasty three-movement manipulation plus HTPGP or placebo plaster (PLAGP) for 2 weeks, with 4 weeks of follow-up. The primary outcome was pain intensity measured by the visual analog scale (VAS); secondary outcomes included the Oswestry Disability Index (ODI) and surface electromyography (sEMG). Analyses were performed using generalized estimating equations, repeated-measures ANOVA, and Mann–Whitney U tests.

**Results:**

Between October 2024 and June 2025, 100 eligible participants were randomized (HTPGP vs. PLAGP, 1:1). The HTPGP group demonstrated significantly greater VAS improvement from day 7 onwards (Z = 2.644–4.332). ODI scores were consistently lower in the HTPGP group (main effect F = 33.43; time effect F = 227.21; interaction F = 17.19). No significant between-group differences were observed in sEMG outcomes, and no serious adverse events occurred.

**Conclusion:**

In patients with LDH, HTPGP combined with manipulation provided greater pain reduction and better functional improvement than those observed in the placebo group, with a favorable safety profile. Larger randomized trials are required to confirm these findings.

**Trial registration:**

The trial was registered with the International Traditional Medicine Clinical Trial Registry (http://itmctr.ccebtcm.org.cn/; No. ITMCTR2024000077).

## Graphical Abstract

**Figure:**
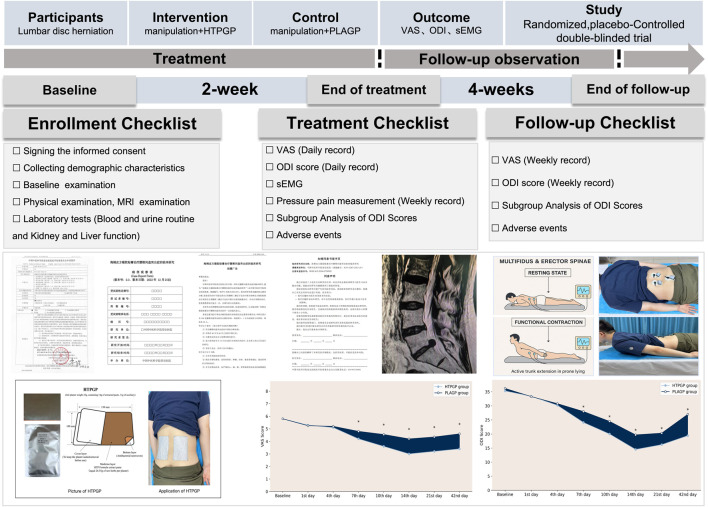

## 1 Introduction

Lumbar disc herniation (LDH) is one of the most common degenerative diseases of the lumbar spine and is closely associated with aging (Pojskic et al., 2024). Its global prevalence is estimated at 1%–5%, with higher rates observed among male manual laborers and smokers (Shiga, 2022). Recently, the incidence has been rising among young adults due to increasingly sedentary lifestyles (Hincapié et al., 2025). LDH not only impairs patients' quality of life and work capacity but also leads to substantial medical resource use and socioeconomic burden (GBD, 2019 Diseases and Injuries Collaborators, 2020). Chronic low back pain caused by LDH has become a major contributor to workforce loss and disability. In China, direct medical costs and indirect losses associated with LDH account for a significant proportion of the economic burden of musculoskeletal disorders (Fan et al., 2023). Consequently, early intervention and effective treatment for LDH have become a priority in clinical practice and public health worldwide.

The main clinical manifestations of LDH are low back pain, radiating pain, numbness in the lower extremities, and limited lumbar mobility (Kreiner et al., 2014). The pathogenesis reflects a degenerative cascade within the intervertebral disc. With aging and increased loading, proteoglycans and water are lost from the nucleus pulposus, accompanied by annular tears that facilitate protrusion through the compromised annulus fibrosus (The pathogenesis reflects a degenerative Cascade within the intervertebral disc, 2025). Beyond mechanical compression of nerve roots, the extruded nucleus pulposus triggers intense inflammatory responses (e.g., TNF-α, IL-1β, prostaglandins) and matrix remodeling, promoting neovascularization within the annulus fibrosus and neurogenic pain (Binch et al., 2014). Neuro-immune interactions and dorsal root ganglion sensitization further exacerbate radicular pain and sensory abnormalities (Wojdasiewicz et al., 2020). Clinically, pain management for LDH remains a significant challenge. LDH leads to substantial consumption of medical resources (imaging, injections, surgery) and is associated with high indirect costs and a high risk of recurrence (GBD, 2021 Low Back Pain Collaborators, 2023). A prospective cohort study reported no significant differences in visual analog scale (VAS) and Oswestry Disability Index (ODI) scores between nonsurgical and surgical treatments over 24 months of follow-up (Kim et al., 2021). A systematic review reported that conservative treatment is less effective than microdiscectomy in the short term but yields similar long-term outcomes (Wang et al., 2020; Thackeray et al., 2017; Hahne et al., 2010). Accordingly, conservative treatment remains the preferred option in clinical practice guidelines in mainland China (Spinal Surgery Group et al., 2020). However, LDH symptoms are complex, prone to recurrence, and often require prolonged conservative management. Therefore, there is growing interest in developing safe and effective topical agents as supplements to nonsurgical therapies.

Nonsurgical treatments are often considered the preferred initial approach, and many have been shown to reduce pain and improve lumbar function in low-risk patients (El Melhat et al., 2024). In China, topical preparations of traditional Chinese medicine (TCM) are a well-established form of external therapy. Herbal plasters are widely preferred by patients for their convenience and efficacy in pain relief (Zhao et al., 2021). Gel plasters, also known as babu plasters, are defined in the 2015 edition of the *Chinese Pharmacopoeia* as a subtype of plasters (Cao et al., 2024). They are characterized by systemic pharmacological effects that do not require oral intake or injection and are associated with fewer adverse reactions. Another advantage of gel pastes is their high drug-loading capacity, which can exceed 20% for herbal powders or complex extracts (Zhang et al., 2023), making them an effective carrier for Chinese medicine prescriptions. Haitongpi formula from the *YiZong JinJian* is a classic topical prescription in TCM. Previous studies have demonstrated its efficacy and safety in treating various musculoskeletal disorders (Li et al., 2025). However, its traditional form was inconvenient for use, which led to the development of Haitongpi Formula Gel Paste (HTPGP). Although HTPGP offers a novel therapeutic option for LDH, its clinical application has been limited by the scarcity of randomized controlled trials (RCTs). We hypothesized that HTPGP would be effective in relieving low back pain and improving function. Additionally, we aimed to evaluate the clinical efficacy and safety of HTPGP combined with manipulation in relieving pain and improving function in patients with LDH.

## 2 Materials and methods

### 2.1 Study design

This was a prospective, double-arm, randomized, double-blind RCT conducted at the Wangjing Hospital of the China Academy of Chinese Medical Sciences (CACMS), the National Center for Orthopedic and Traumatic Diseases in China, between October 2024 and June 2025. The study adhered to the Consolidated Standards of Reporting Trials (CONSORT) 2010 Statement (Schulz et al., 2010), which standardizes guidelines for reporting clinical trials.

The study screened 100 patients with LDH aged over 40 years using magnetic resonance imaging (MRI) and clinical examination, including baseline assessment with VAS scores. Eligible participants were randomly assigned in a 1:1 ratio to receive either manipulation combined with HTPGP or manipulation combined with PLAGP. Each group received manipulation once every 3 days and topical medication five doses per week. The primary outcome measure was the VAS, and secondary outcomes included the ODI and surface electromyography (sEMG). Participants completed seven visits: baseline screening at enrollment, assessments on days 1, 4, 7, 10, and 14, with day 14 marking the end of manipulative therapy, and follow-up assessments on days 21 and 42, which were conducted either in person, online, or by telephone. Additionally, sEMG of the lumbar muscles was performed by a spine surgeon at baseline and after 2 weeks of therapy (day 14). Each patient received 2 weeks of treatment followed by a 4-week follow-up period. Participants were informed that they could withdraw from the study at any time without consequences.

### 2.2 Recruitment, inclusion, and exclusion criteria

All participants were recruited through posters and outpatient visits at Wangjing Hospital. At the screening stage, experienced clinicians evaluated eligibility based on clinical symptoms, imaging findings (lumbar MRI or CT), and a comprehensive review of the inclusion and exclusion criteria.

The diagnostic criteria for LDH were based on the *Chinese Pain Expert Consensus on the Treatment of Lumbar Disc Herniation* (Spinal Surgery Group et al., 2020). Participants were required to have LDH confirmed by MRI or CT, with imaging findings consistent with clinical symptoms. Eligibility was established if three or more of items (a–e) were present in combination with item (f): a) Radiating pain in the lower limbs consistent with the innervation area of the affected nerves; b) Sensory abnormality in the lower limbs, with reduced superficial sensation in the corresponding innervation area; c) Positive straight-leg-raising test, reinforcement test, contralateral straight-leg-raising test, or femoral nerve stretch test; d) Weakened tendon reflexes compared to the healthy side; e) Decreased muscle strength; and f) Lumbar MRI or CT confirming disc herniation with nerve compression consistent with the patient’s symptoms and signs.

Inclusion criteria were as follows: a) Participants aged 40–60 years with typical clinical symptoms of low back pain and a VAS score ≤7; b) Confirmed diagnosis of LDH by imaging and clinical assessment; c) Voluntary agreement to participate in the trial and provision of written informed consent in compliance with the Good Clinical Practice (GCP) regulations. Exclusion criteria included: a) History of spinal infections, traumatic fractures, tumors, tuberculosis, severe osteoporosis, ankylosing spondylitis, or other diseases that may affect study outcomes; b) Contraindications to physical therapy, such as severe organic diseases of the heart, brain, lungs, or kidneys, or pregnancy/lactation; c) Contraindications to topical medication, including local skin damage or open wounds; d) Acute stage of disease with a VAS pain score >7; e) Known drug allergies or a history of severe allergic reactions to medications; f) Concomitant use of medications for other diseases that may interfere with study outcomes; g) Participation in other clinical trials during the study period or refusal to provide informed consent.

### 2.3 Randomization and blinding

Randomization and blinding were managed by the Institute of Basic Research in Clinical Medicine, CACMS, independent of the study investigators. Eligible participants who provided written informed consent were randomly assigned in a 1:1 ratio to receive either HTPGP or PLAGP. The assigned intervention (HTPGP or placebo) was disclosed only to pharmacists, who were not involved in outcome evaluation or data analysis. This was a double-blind study: neither participants nor investigators were aware of group assignments, and the placebo was indistinguishable from the active treatment in appearance, smell, and packaging.

### 2.4 HTPGP and placebo preparation

**FIGURE 1:**
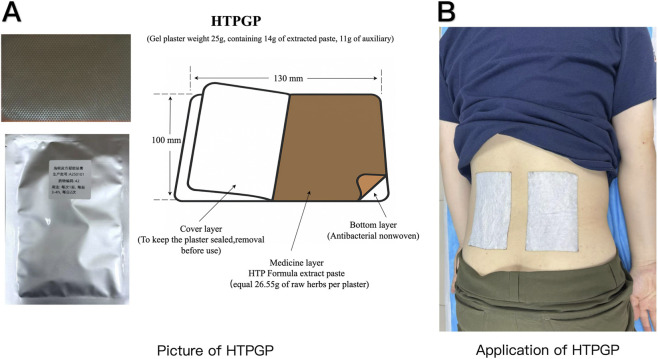
The layer of the HTPGP **(A)** and Applications of HTPGP **(B)**.

The HTPGP was prepared under Good Manufacturing Practice (GMP) standards in collaboration with the Institute of Chinese Materia Medica, CACMS, following the *Technical Guidelines for the Preparation of Drugs for Clinical Trials of New Chinese Medicines* issued by the Center for Drug Evaluation, National Medical Products Administration. Preparation and quality control of HTPGP and PLAGP complied with both *GMP and GCP requirements for clinical use*. Table 1 describes the herbs, their botanical names, families, harvest locations and seasons, and processing methods. Details of HTPGP, PLAGP and herbal material quality certification are provided in Supplementary Material S1. Details of the transdermal components and herbal chemical constituents of HTPGP are available in Supplementary Material S2. The structural layers and applications of HTPGP are shown in Figure 1.

**TABLE 1:** Herbal ingredients of Haitongpi formular gel plaster.

Herbs	Botanical name	Family	Harvesting place	Harvesting season	Processing method
Z. ailanthoides bark (海桐皮)	Zanthoxylum ailanthoides sieb. and Zucc	Rutaceae	Zhejiang, China	Autumn	Bark peeled in autumn, cleaned, dried in the sun or shade, and cut into pieces before decoction
Clematis intricata (铁线透骨草)	Clematis intricata bunge	Ranunculaceae	Hebei, China	Summer (July–August)	Whole plant harvested in summer, cleaned, dried, and cut into pieces
Frankincense (乳香)	Boswellia sacra flueck./B. carterii birdw	Burseraceae	Ethiopia	June – September	Resin collected, cleaned, dried in shade, used whole or crushed, and vinegar-processed before use
Myrrh (没药)	Commiphora myrrha (nees) Engl	Burseraceae	Kenya	November–March	Resin collected, sun-dried, impurities removed, crushed for decoction, and vinegar-processed before use
Angelica sinensis (酒当归)	Angelica sinensis (oliv.) diels	Apiaceae	Gansu, China	October–November	Roots harvested, fibrous roots removed, sliced, and wine-fried before use
Sichuan pepper (花椒)	Zanthoxylum bungeanum maxim	Rutaceae	Sichuan, China	July–September	Fruits harvested at ripening, dried in the sun, stalks and seeds removed, and used raw
Chuanxiong (川芎)	Ligusticum chuanxiong hort	Apiaceae	Sichuan, China	Mid-April–End of May	Rhizomes harvested, cleaned, boiled briefly, sun-dried, sliced before decoction
Safflower (红花)	Carthamus tinctorius L	Asteraceae	Xinjiang, China	September–October	Flowers picked at early bloom, quickly dried in shade or sunlight, then used raw, whole, or ground
Clematis chinensis osbeck (威灵仙)	Clematis chinensis osbeck	Ranunculaceae	Anhui, China	Autumn	Roots dug in autumn, washed, dried, and cut into sections
Dahurian angelica (白芷)	Angelica dahurica (hoffm.) benth. and Hook.f. ex franch. and sav	Apiaceae	Sichuan, China	Summer–Autumn	Roots collected post-flowering, cleaned, boiled briefly, dried, and sliced
Fang feng (防风)	Saposhnikovia divaricata (turcz.) schischk	Apiaceae	Heilongjiang, China	Early spring or late autumn	Roots harvested, fibrous roots and root head removed, sun-dried, and sliced
Licorice root (甘草)	Glycyrrhiza uralensis fisch	Fabaceae	Nei mongol, China	August–October	Roots collected in autumn, washed, dried, and cut into slices or segments

### 2.5 Interventions

#### 2.5.1 Manipulation therapy

Orthopedic manipulation techniques in TCM, an indispensable component of nonoperative treatments for LDH, are effective and widely used in China (Mo et al., 2018). The three-step oblique pulling spinal manipulation, originating from the Orthopedic Manipulation Techniques of the Qing Imperial Court, represents a typical manual therapy approach. Detailed procedural steps are provided in Supplementary Material S3.

#### 2.5.2 Application of the HTPGP and PLAGP

In both the HTPGP and PLAGP groups, a rectangular plaster (100 × 140 mm) was applied to the lower back at the site of pain (Figure 1). Each plaster was worn for 4 h per day for 5 consecutive days, followed by a two-day break to minimize skin irritation. Concurrently, patients received manipulation therapy once every 3 days. This cycle was continued for 2 weeks, for a total of five sessions. Any modifications to the treatment regimen were documented, and no alterations to potentially disease-modifying therapies were permitted.

### 2.6 Sample size

Given the lack of published RCTs of HTPGP versus placebo for LDH, the sample size was estimated based on a previous clinical trial of the HaiTongPi formula (Li et al., 2025) and additional indirect evidence (Shang et al., 2022). The primary outcome was the VAS score after 2 weeks of treatment. A reduction in VAS score of at least 2 points or ≥50% was considered clinically meaningful. Assuming a two-sided significance level of 5% (α = 0.05) and 90% power (β = 0.10), 41 patients per group were required. To account for a 20% dropout rate, the sample size was increased to 50 patients per group, resulting in a total enrollment target of 100 patients.

### 2.7 Primary outcomes

The primary outcome was the VAS score, ranging from 0 to 10, with higher scores indicating greater pain intensity. Clinicians assessed patients before each manipulation session (baseline, on days 1, 4, 7, 10, and 14) and during the follow-up (days 21 and 42).

### 2.8 Secondary outcomes

Secondary outcomes included the ODI (Roland and Fairbank, 2000) and sEMG of the multifidus and erector spinae muscles bilaterally. The ODI is a widely used tool for assessing low back pain and functional status (Liu et al., 2009; Fritz and Irrgang, 2001). It assesses 10 dimensions: pain, personal care, lifting, walking, sitting, standing, sleeping, sex life, social life, and traveling. Each item is scored on a 6-point Likert scale (0–5), with higher scores reflecting greater disability (Ruiz et al., 2014). Similar to the VAS, ODI assessments were conducted by clinicians through patient interviews before each manipulation session.

sEMG is a noninvasive technique for recording muscle electrical activity and is widely used to assess muscle fatigue and monitor therapeutic effects (Mohseni Bandpei et al., 2014). Commonly analyzed parameters include median frequency (MF), mean power frequency, and root mean square (RMS) (Neblett, 2016). Signals were analyzed in both the time and frequency domains. RMS, a time-domain index, reflects the intensity of muscle activation and neural recruitment by averaging squared signal amplitudes after rectification and smoothing (Clancy et al., 2023). MF, a frequency-domain parameter, is calculated using the fast Fourier transform (FFT) of the power spectral density curve and represents the frequency at which half of the signal’s total energy is reached (Wang et al., 2016). Elevated RMS values usually indicate increased muscle activation, while a decline in MF is considered a sensitive marker of muscle fatigue. Combining RMS and MF provides a comprehensive assessment of muscle function, therapeutic effects, and fatigue changes during rehabilitation (Blanco-Giménez et al., 2024).

sEMG of the multifidus and erector spinae muscles was recorded at baseline and day 14. RMS (%MVC) and MF were calculated to assess muscle function and fatigue. Detailed acquisition parameters are provided in Supplementary Material S4.

### 2.9 Safety indicators and treatment of adverse events

An adverse event reporting system was established to record any adverse reactions occurring during the trial, including the time of onset, severity, duration, and corresponding management measures. Each adverse event was evaluated for causality and its potential association with the study intervention. If lumbar or leg pain worsened following manipulation therapy, the intensity and duration of the technique were adjusted according to the patient’s tolerance and individual responses. In case of severe allergic reaction to the intervention, treatment was immediately discontinued, and the event was recorded in detail. A comprehensive physical examination was conducted, and a dermatologist was consulted to provide professional evaluation and management.

### 2.10 Statistical analysis

All statistical analyses were performed using SPSS software (version 26.0), with additional model validation and graphing conducted in R (version 4.3.2). To assess treatment efficacy over time and differences between groups at multiple time points, appropriate statistical methods were applied based on the data characteristics. Results were interpreted not only in terms of statistical significance but also in line with the CONSORT statement, with attention to clinical significance. The minimum clinically important difference (MCID) was used to determine whether participants achieved patient-perceivable improvement, with an MCID of 2.0 points established for VAS (Ostelo et al., 2008).

Missing data: Data collection during the treatment phase was highly complete due to the supervised recording at each therapy session. Two participants did not complete the Allergy and Adverse Event Reporting sections of the case report form; however, no clinical symptoms or spontaneous reports were recorded during follow-up. Therefore, these cases were not considered adverse events, and the missing data were deemed unlikely to affect the completeness or validity of the statistical analysis.

Normality of continuous variables was assessed using the Shapiro–Wilk test. The ODI total scores approximates a normal distribution, whereas the VAS, ODI subscales, and sEMG data all exhibit non-normal distributions. Different statistical analysis methods were selected based on the specific characteristics of each data distribution. For VAS scores, generalized estimating equations were used to analyze the intervention effects, accounting for correlations in repeated measures, non-normality, and missing follow-up data. The statistical significance of the group × time interaction term was considered as the primary indicator of the intervention effect. For ODI total scores, repeated measures analysis of variance (RM-ANOVA) was applied to evaluate the effects of time, group, and their interactions. When significant interaction effects were observed, *post hoc* pairwise comparisons were conducted using Bonferroni correction. ODI subscales were analyzed using the Mann–Whitney U test. sEMG data, expressed as normalized RMS (%MVC) and MF, were compared between groups using nonparametric tests. Adverse event rates and MCID achievement were analyzed using the chi-squared test or Fisher’s exact test, as appropriate. All tests were two-sided, and statistical significance was set at *P* < 0.05.

## 3 Results

**FIGURE 2:**
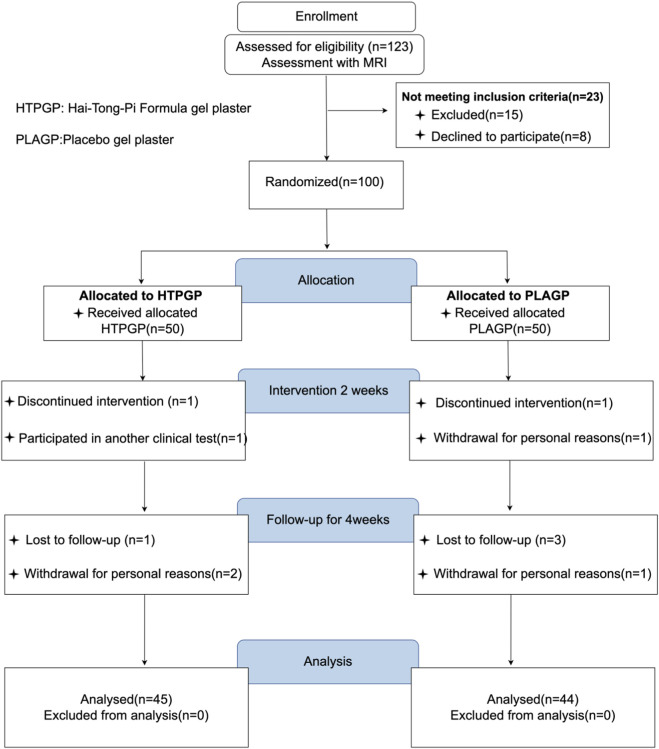
Consolidated Standards of Reporting Trials (CONSORT) flow chart of the HTPGP trial.

Figure 2 shows the screening process for all participants. Between October 2024 and June 2025, 123 participants were screened and assessed for eligibility. Fifteen participants were excluded because they did not meet the inclusion or exclusion criteria. Of the remaining 108 participants, 100 were enrolled in the trial, whereas 8 declined to participate. During data validation, five participants from the HTPGP group and six from the PLAGP group were excluded. Finally, 45 participants in the HTPGP group and 44 in the PLAGP group were included in the analysis.

### 3.1 Baseline characteristics

Baseline characteristics were comparable between the two groups, with no statistically significant differences observed. Both groups were predominantly women (55.06%), and body mass index (BMI) and vital signs were within the normal ranges. All participants had a long disease duration, and most had received one or more prior treatments, including oral analgesics, topical medications, acupuncture, and physical therapy. Regarding comorbidities, 65.17% of participants had no comorbidities, 26.97% had a history of hypertension, and 11.24% had type 2 diabetes. Demographic and clinical characteristics are summarized in Table 2.

**TABLE 2:** Baseline characteristics of the participants.

Outcomes				HTPGP group (n = 45)	PLAGP group (n = 44)	P value
Woman			n (%)	24 (53.33)	25 (56.82)	​
Age (years)			Mean ± SD	50.62 ± 4.53	49.91 ± 4.76	0.471
BMI, mean (kg/m2)			Mean ± SD	23.25 ± 0.59	23.21 ± 0.57	0.706
Temperature (°C)			Mean ± SD	36.51 ± 0.25	36.49 ± 0.23	0.629
Pulse (cpm)			Mean ± SD	75.29 ± 3.86	76.59 ± 4.02	0.123
Respiration (cpm)			Mean ± SD	15.93 ± 2.19	16.95 ± 2.08	0.780
Blood pressure (mmHg)						
Systolic			Mean ± SD	124.29 ± 4.94	125.43 ± 5.19	0.290
Diastolic			Mean ± SD	81.69 ± 4.64	82.64 ± 4.68	0.340
Duration of disease (d)			Mean ± SD	1029.11 ± 744.14	1188.07 ± 917.40	0.371
Smoking			n (%)	12 (26.67)	14 (31.82)	0.593
Drinking			n (%)	8 (17.78)	7 (15.91)	0.814
Primary therapy						
Oral analgesic			n (%)	14 (31.11)	11 (25.00)	0.521
Acupuncture			n (%)	21 (46.67)	18 (40.91)	0.584
Physical therapy			n (%)	10 (22.22)	12 (27.27)	0.581
Topical medication			n (%)	36 (80.00)	38 (86.36)	0.422
No specific therapy			n (%)	4 (8.89)	6 (13.64)	0.522
Comorbidities						
Hypertension			n (%)	10 (22.22)	14 (31.82)	0.308
Type II diabetes			n (%)	6 (13.33)	4 (9.09)	0.739
Hyperlipidemia			n (%)	3 (6.67)	1 (2.27)	0.616
Cardiovascular disease			n (%)	3 (6.67)	1 (2.27)	0.616
None			n (%)	31 (68.89)	27 (61.36)	0.456
Marital state						
Married			n (%)	38 (84.44)	41 (93.18)	0.315
Unmarried			n (%)	1 (2.22)	0 (0)	1.000
Divorced			n (%)	2 (4.44)	1 (2.27)	1.000
Widowed			n (%)	4 (8.89)	2 (4.55)	0.677
Educational background						
Secondary school or below			n (%)	16 (35.56)	14 (31.82)	0.709
Undergraduate			n (%)	26 (57.78)	25 (56.82)	0.927
Postgraduate			n (%)	3 (6.67)	5 (11.36)	0.485
VAS			Mean (95%CI)	5.78 (5.38, 6.16)	5.80 (5.41, 6.19)	0.915
ODI			Mean (95%CI)	35.40 (33.98, 36.82)	36.14 (34.70, 37.57)	0.471
sEMG						
Erector spinae muscles	Left	MF(Hz)	Mean (IQR)	64.16 [61.10, 68.79]	62.76 [58.49, 65.76]	0.337
		RMS(µV)	Mean (IQR)	39.26 [35.81, 44.25]	37.50 [31.18,44.54]	0.201
	Right	MF (Hz)	Mean (IQR)	63.47 [58.59, 70.05]	63.37 [53.09, 69.08]	0.333
		RMS(µV)	Mean (IQR)	38.85 [34.29, 42.94]	38.68 [32.63,45.11]	0.694
Multifidus muscles	Left	MF(Hz)	Mean (IQR)	64.64 [59.94, 68.72]	64.36 [60.01,69.02]	0.915
		RMS(µV)	Mean (IQR)	41.49 [36.48, 44.85]	40.92 [37.45, 45.10]	0.679
	Right	MF(Hz)	Mean (IQR)	64.06 [58.80, 67.89]	63.54 [55.84, 68.65]	0.931
		RMS(µV)	Mean (IQR)	40.80 [35.65, 45.04]	38.83 [33.16,43.96]	0.360

### 3.2 Primary outcomes

There was no significant difference in baseline VAS scores between the two groups (group main effect: *Z* = 0.107, *P* = 0.915), and both groups showed significant decreases over time. The HTPGP group exhibited significantly greater improvement than that of the PLAGP group from day 7 onwards (group-by-time interaction effect: day 7: *Z* = 2.644, *P* = 0.008; day 10: *Z* = 3.472, *P* = 0.001; day 14: *Z* = 4.332, *P* < 0.001; day 21: *Z* = 3.983, *P* < 0.001; day 42: *Z* = 3.517, *P* < 0.001). MCID analysis showed that the HTPGP group achieved a significantly higher rate of VAS improvement from day 7 than that of the PLAGP group. On day 7, responder rates were 48% in the HTPGP group and 14% in the PLAGP group (*P* = 0.001; OR = 5.69, 95% CI: 2.04–16.34). On day 10, rates were 59% versus 25% (*P* = 0.002; OR = 4.35, 95% CI: 1.68–11.56); on day 14, 80% versus 45% (*P* = 0.002; OR = 5.11, 95% CI: 1.82–15.34); on day 21, 77% versus 36% (*P* < 0.001; OR = 6.23, 95% CI: 2.32–17.45); and on day 42, 64% versus 30% (*P* = 0.003; OR = 4.09, 95% CI: 1.61–10.45). These results suggest that HTPGP has clear analgesic efficacy, with a rapid onset of action that persisted throughout the follow-up period.

### 3.3 Secondary outcomes

#### 3.3.1 ODI

Repeated-measures ANOVA showed a significant group main effect (*F* = 33.43, *P* < 0.001), with the HTPGP group consistently exhibiting lower mean ODI values than those for the placebo group. Both the time main effect and the group-by-time interaction effect were also significant (*F* = 227.21, *P* < 0.001; *F* = 17.19, *P* < 0.001), indicating an overall decrease in ODI scores over time and greater improvement in the HTPGP group than in the PLAGP group. The trends of VAS and ODI score reductions for both groups are presented in Table 3.

**TABLE 3:** Study outcomes across study time points.

Time	HTPGP group (n = 45)	PLAGP group (n = 44)	P value
	Mean (95%CI)	Mean (95%CI)	
VAS			
Baseline	5.77 (5.38, 6.16)	5.80 (5.41, 6.19)	0.915
1st day	5.30 (4.96, 5.64)	5.27 (4.94, 5.60)	0.824
4th day	5.09 (4.79, 5.39)	5.22 (4.89, 5.55)	0.393
7th day	4.16 (3.84, 4.48)	4.83 (4.48, 5.18)	<0.01
10th day	3.62 (3.24, 4.00)	4.54 (4.15, 4.93)	<0.01
14th day	2.99 (2.65, 3.33)	4.13 (3.77, 4.49)	<0.001
21st day	3.20 (2.83, 3.57)	4.28 (3.94, 4.62)	<0.001
42nd day	3.34 (2.94, 3.74)	4.49 (4.07, 4.91)	<0.001
ODI			
Baseline	35.40 (33.98, 36.82)	36.14 (34.70, 37.57)	0.471
1st day	33.27 (31.84, 34.70)	33.23 (31.78, 34.67)	0.966
4th day	30.22 (28.79, 31.66)	30.91 (29.46, 32.36)	0.452
7th day	24.04 (22.54, 25.55)	28.27 (26.75, 29.80)	<0.001
10th day	20.07 (18.56, 21.57)	24.52 (23.00, 26.04)	<0.001
14th day	14.51 (13.64, 15.39)	19.66 (18.77, 20.54)	<0.001
21st day	16.29 (15.35, 17.23)	20.18 (19.23, 21.13)	<0.001
42nd day	19.33 (18.44, 20.23)	26.80 (25.95, 27.64)	<0.001

**FIGURE 3:**
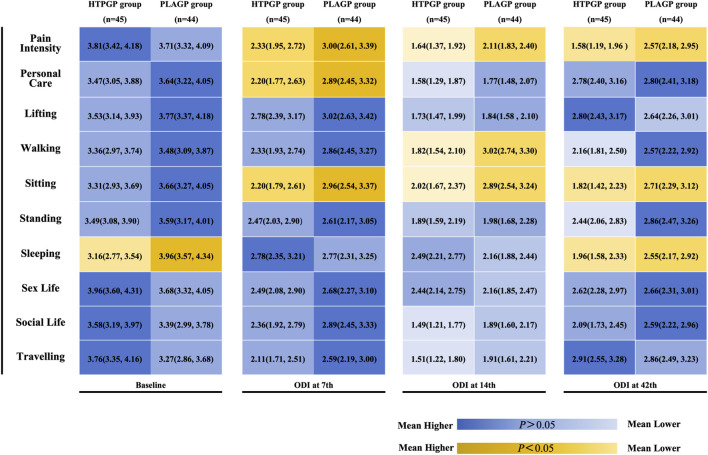
Subgroup analysis of ODI scores by time point.

**FIGURE 4:**
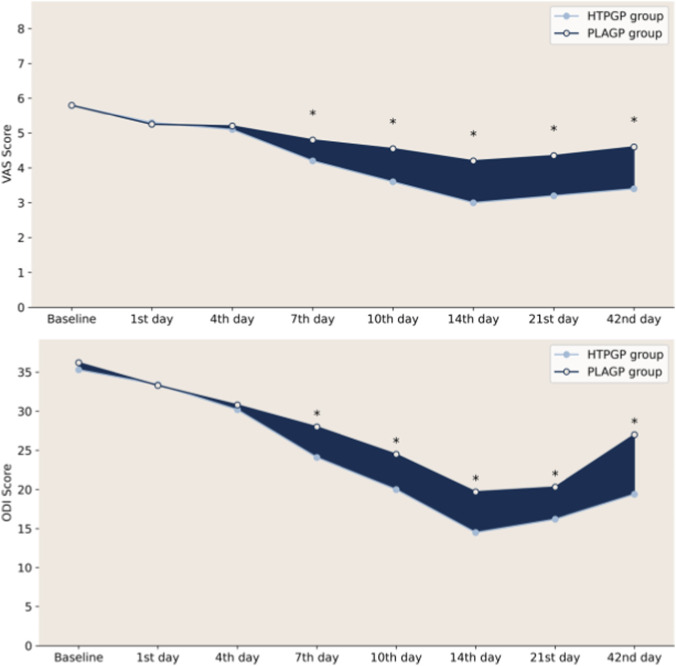
Difference plots of VAS and ODI scores at each follow-up point.

Group comparisons of the 10 functional subscales of the ODI at each time point were also performed, and the results are shown in Figure 3, with color-block labeling to highlight statistical differences and mean values. Yellow indicates a significant difference between the two groups, whereas blue represents no significant difference. Color intensity reflects the mean values of each group (darker colors indicate higher mean values, suggesting more severe dysfunction). At baseline, there were no significant differences in most individual ODI scores between the two groups in terms of pain, personal care, or function (*P* > 0.05). After 1 week of treatment (day 7), the HTPGP group showed significantly lower scores than those of the PLAGP group in the pain, personal care, and sitting subscales (*P* < 0.05). After 2 weeks of treatment (day 14) and at the 4-week follow-up, the HTPGP group had significantly lower scores than those of the PLAGP group on several subscales, including pain and sitting (*P* < 0.05). HTPGP improved pain and functional scores in the ODI subscales for patients with LDH and demonstrated greater efficacy than that of PLAGP. Trends in VAS and ODI intergroup comparisons are shown in Figure 4.

#### 3.3.2 sEMG

sEMG values were collected from participants in both groups before and after the intervention to obtain MF and RMS values for each participant. These values were normalized to percentages using the formula: (post–pre)/pre × 100%. The rate of change (Δ%) in the primary outcomes, MF and RMS, was compared between groups. The results showed that the overall rate of change was similar in the HTPGP and PLAGP groups, with no statistically significant difference (*P* > 0.05). The results are summarized in Table 4.

**TABLE 4:** sEMG outcomes.

Subjects			HTPGP group (n = 45)	PLAGP group (n = 44)	P value
Multifidus	MF	Left	−0.10 [-0.82, 0.50]^*^	−0.28 [-0.71, 0.54]	0.810
		Right	−0.35 [-1.15, 0.28]^*^	−0.30 [-1.28, 0.10]	0.447
	RMS	Left	0.01 [-0.49, −0.84]^*^	0.23 [-0.44, 0.95]	0.560
		Right	−0.35 [-1.19, 0.62]^*^	−0.23 [-1.08, 0.34]	0.763
Erector	MF	Left	−0.45 [-1.18, 0.20]^*^	−0.57 [-1.08, 0.02]	0.655
		Right	−0.06 [-0.78, 0.53]^*^	−0.47 [-1.14, 0.57]	0.212
	RMS	Left	−0.33 [-1.59, 0.76]^*^	−0.60 [-1.31, 0.42]	0.761
		Right	0.61 [-0.50,1.36]^*^	0.23 [-0.79, 0.80]	0.168

Abbreviations: sEMG, surface electromyography; MF, median frequency; RMS, root mean square.

All sEMG, data are expressed as percent change relative to baseline (calculated per subject, per muscle, per side). Values are presented as median (interquartile range). Between-group differences were assessed using the Mann–Whitney U test. *: *P* > 0.05 for all comparisons (not significant).

### 3.4 Safety assessment

Three participants experienced adverse events during the observation period: one in the HTPGP group (skin itching) and two in the PLAGP group (skin flushing). The overall incidence of adverse events was 3.37%, with 2.22% in the HTPGP group and 4.55% in the PLAGP group, with no statistically significant difference between groups (*P* > 0.05).

## 4 Discussion

Pain management in patients with LDH remains a clinical challenge. LDH often follows a prolonged course, is prone to recurrence, and substantially impairs patients' quality of life (Beall et al., 2024). This randomized, double-blind, placebo-controlled trial evaluated the efficacy of topical herbal plasters in the treatment of LDH. The primary efficacy outcome was the VAS score, a widely used and reliable tool for assessing pain. In this study, VAS scores in the HTPGP group decreased significantly and were consistently lower than those in the PLAGP group starting from the 7th day of treatment. At the 4-week follow-up, the HTPGP group continued to show greater improvement, suggesting a clear short-term analgesic effect. Secondary outcomes included the ODI score and sEMG. The ODI, a validated measure for assessing lumbar pain and functional status, also exhibited greater improvement in the HTPGP group than in the PLAGP group from the 7th day onward. Subgroup analysis revealed significant improvements in pain, sitting, standing, and walking scores, whereas other functional domains showed no significant differences. These findings suggest that while HTPGP may not fully restore lumbar function, its analgesic effects likely contribute to functional improvements in activities such as sit-to-stand transitions and walking.

Regarding the MF and RMS values of the sEMG, we expressed the raw values as percentages to account for individual differences. The results showed a trend of improvement in some participants in the HTPGP group; however, the overall difference was not statistically significant. This may be related to the limited sample size, individual variability in muscle structure and innervation, and the time lag between pain relief and muscle function recovery. Pain improvement often precedes full recovery of muscle function, a pattern that has also been reported in rehabilitation studies of other musculoskeletal pain disorders (Neblett, 2016). Therefore, although the changes in sEMG parameters in this study did not demonstrate significant improvements in muscle function, the potential positive effects of HTPGP on muscle function recovery during long-term rehabilitation cannot be excluded.

HTPGP is produced by incorporating Chinese medicines into gel paste excipients, which are prepared in accordance with the provisions of the *Chinese Pharmacopoeia 2020* for gel pastes. The formula contains 12 medicinal components, classified according to the *Chinese Pharmacopoeia 2020*. Among them, four are wind-dampness–removing medicines (Haitongpi, Clematis intricata Bunge, Weilingxian, and Fangfeng); five are blood circulation–promoting medicines (Frankincense, Myrrh, Chuanxiong, Safflower, and Angelica sinensis); Sichuan Pepper is categorized as warming the interior; Dahurian Angelica as relieving the cold; and Licorice Root as supplementing deficiency. The therapeutic principle of this formula is to dispel wind and dampness, promote blood circulation, remove blood stasis, warm the channels and collaterals, and relieve pain and paralysis.

Pharmacological studies have identified active ingredients and mechanisms of action for the components of this formula, which collectively provide anti-inflammatory, analgesic, microcirculatory, and neuroprotective effects. Zanthoxylum ailanthoides bark is rich in alkaloids and flavonoids that exert anti-inflammatory and analgesic effects while inhibiting nerve conduction, thereby reducing peripheral nociceptive signaling (Zhang et al., 2017; Yuan et al., 2021). Clematis intricata Bunge contains triterpenoid saponins and alkaloids with similar anti-inflammatory and analgesic properties (Chawla et al., 2012). Frankincense and Myrrh act synergistically, as both possess well-documented anti-inflammatory and analgesic activities. Frankincense extracts demonstrate anti-inflammatory effects in various acute and chronic inflammatory conditions, likely through the selective inhibition of 5-lipoxygenase (5-LO)–mediated biosynthesis (Catanzaro et al., 2015). Myrrh contains several bioactive anti-inflammatory compounds. Kimura et al. (Kimura et al., 2001) reported that manumbinoic acid, a triterpenoid isolated from Commiphora incisa resin, exhibits anti-inflammatory potency comparable to that of indomethacin and prednisolone (Cao et al., 2019). Additionally, Mehta et al. demonstrated that Indian myrrh extract significantly alleviates thermal hyperalgesia and abnormal cold pain (Mehta and Tripathi, 2015). The combined use of frankincense and myrrh synergistically enhances anti-inflammatory, analgesic, antibacterial, and blood circulation–promoting effects. Chuanxiong and Safflower can improve microcirculatory blood flow, inhibit platelet aggregation, and reduce oxidative stress, thereby alleviating ischemic nerve injury (Wang et al., 2025; Shi et al., 2020; Mani et al., 2020; Cheng et al., 2024). Angelica sinensis exerts anti-inflammatory, neuroprotective, and tissue repair effects through its polysaccharide, ferulic acid, and volatile oil components. Wei Ling Xian, Angelica dahurica, and Fang Feng contribute to analgesia by modulating the release of inflammatory mediators and relieving muscle spasms. These synergistic pharmacological effects have been confirmed in recent experimental studies (Wei et al., 2016; Nai et al., 2021).

During HTPGP preparation, volatile oils were individually extracted from the herbs containing these components. In addition to their pharmacological activities, these volatile oils also promote transdermal absorption. The frankincense–myrrh complex has been shown to exert a significant pro-permeability effect *in vitro* (Guan et al., 2017). Sichuan peppers may activate TRPV1 channels through sanguinarine, inducing a warming sensation, promoting local vasodilation, and thereby enhancing the transdermal permeation of administered drugs (Zhang et al., 2017; Koo et al., 2007; Lennertz et al., 2010). Systematic studies have demonstrated that Chuanxiong oil provides higher transdermal penetration of ibuprofen than that provided by the chemical permeation enhancer Azone and produces a superior analgesic effect in a mouse model of dysmenorrhea. This effect may be mediated by the perturbation of stratum corneum lipids, increased lipid mobility, and improved microcirculation (Chen et al., 2015). Additionally, volatile oils derived from Angelica sinensis and Angelica dahurica have also been shown to significantly promote transdermal drug delivery (Lu and He, 2003; Lin et al., 2018).

Considering that HTPGP is administered transdermally, we performed compositional identification and transdermal compositional analysis of HTPGP using UHPLC-Q-Orbitrap HRMS to clarify its mechanism of action and strengthen clinical evidence. The composition of HTPGP was identified in the paste prior to application, and the transdermal constituents were determined in the subcutaneous tissues of rats after 14 days of transdermal administration. The identified active ingredients and transdermal constituents are consistent with the pharmacological effects of the herbs described in the previous section; therefore, they are not further discussed here.

In summary, HTPGP may exert synergistic analgesic effects in patients with LDH through multiple targets and pathways. Transdermal administration allows the active ingredient to reach the site of local pain, achieving high local concentrations while minimizing systemic exposure. Its anti-inflammatory effects may reduce peripheral sensitization, whereas improved microcirculation and neuroprotection may facilitate functional recovery. These mechanisms may underlie the persistent analgesic effects observed in this study and are consistent with the clinical findings. HTPGP demonstrates clear advantages in providing short-term pain relief and partial functional improvement.

This study has some limitations. First, it was conducted at a single center and should be validated in future multicenter studies. Second, the follow-up period was limited to 6 weeks; long-term trials are required to evaluate sustained efficacy. Third, as all participants experienced low back pain at the time of enrollment, it was not ethical to administer a placebo alone. To ensure symptom relief, we provided basic manipulative therapy. Future studies should compare the present findings with those obtained using an already approved analgesic gel paste.

## 5 Conclusion

HTPGP demonstrated clinical efficacy in this randomized, placebo-controlled trial, as evidenced by improvements in VAS scores, ODI, and sEMG outcomes. No adverse systemic effects were reported. These findings suggest that HTPGP provides pain relief with a favorable safety profile and represents a promising adjunct treatment option for patients with LDH.

## Data Availability

The original contributions presented in the study are included in the article/Supplementary Material, further inquiries can be directed to the corresponding authors.
