# Supplementary material for: Clinical efficacy of a Chinese herbal gel plaster combined with manipulation for lumbar disc herniation: a prospective, randomized, double-blind, placebo-controlled trial
Source: Front Pharmacol. 2026 Mar 19;17:1708694. doi: 10.3389/fphar.2026.1708694 (PMC13044584; doi:10.3389/fphar.2026.1708694)
Supplement: Supplementary file 1 [file Supplementaryfile1.docx]

Supplementary Material

# **SECTION 1**

**HTPGP and placebo preparation**

HTPGP consisted of 51 g of herbs, including Pittosporum bark, Clematis intricata Bunge, frankincense, myrrh, Angelica sinensis, Sichuan pepper, Chuanxiong, safflower, Clematis chinensis Osbeck, Dahurian angelica, fang feng, and licorice root, in a ratio of 6:6:6:6:5:9:3:3:3:3:3:3.

The herbs were weighed according to these proportions. Frankincense and myrrh were crushed into particles < 0.5 cm in diameter. Angelica sinensis, Sichuan pepper, Chuanxiong, Dahurian angelica, and fang feng were distilled to extract volatile oil, which was stored separately along with the decoction. The dregs of Pittosporum bark, safflower, licorice root, Clematis chinensis Osbeck, and Clematis intricata Bunge were extracted with water. All decoctions were combined and concentrated, followed by alcoholic precipitation with ethanol at room or low temperature. The concentrate was then mixed with volatile oil to obtain the extract paste. The excipients included glycerol, kaolin, glycinaluminum, EDTA-2Na, NP-700, PVPP, polyvinylpyrrolidone K90, nitrogen ketone, propylene glycol, and ethyl hydroxyphenyl ether. The extract paste was blended with the excipients by adding 10% citric acid in water, thoroughly mixed under vacuum, coated with a capsule liner, and sliced to obtain HTPGP.

The placebo plaster was prepared by replacing the herbal extract with edible coloring agents and traditional Chinese herbal fragrances to mimic the color and odor of HTPGP. The same proportion of excipients used in the HTPGP formulation was applied to the placebo, with purified water added to adjust the total weight to match that of the active plaster. The placebo preparation followed the aforementioned guidelines. Both the active and placebo plasters were identical in shape, size, weight, and external presentation, ensuring effective blinding of participants and clinical investigators.

**Quality certificates of the herbal materials**

Pittosporum Bark was obtained from Beijing Bencao Fangyuan Pharmaceutical Group(batch number 20240711, Zhejiang, China). The identity and purity of the compound were confirmed using the Beijing Municipal Code of Concoction of Chinese Medicinal Pieces (2023 edition). The laboratory of Beijing Bencao Fangyuan Pharmaceutical Group conducted residue analysis and organic trace analysis (Beijing, China) (C24-072808) and confirmed that the Impurities, moisture, total ash and sulphur dioxide contamination was within the guidelines of the Beijing Municipal Code of Concoction of Chinese Medicinal Pieces (2023 edition).

Clematis intricata Bunge was obtained from Beijing Jinchongguang Pharmaceutical Co., Ltd. (batch number 2407059, Hebei, China). The identity and purity of the compound were confirmed using the Beijing Municipal Code of Concoction of Chinese Medicinal Pieces (2023 edition). The laboratory of Beijing Jinchongguang Pharmaceutical Co., Ltd. conducted residue analysis and organic trace analysis (Beijing, China) (C202407059) and confirmed that the Impurities, moisture, total ash and sulphur dioxide contamination was within the guidelines of the Beijing Municipal Code of Concoction of Chinese Medicinal Pieces (2023 edition).

Frankincense was obtained from Beijing Sifang Chinese medicine pieces Co., Ltd.(batch number 23042801, Ethiopia).The identity and purity of the compound were confirmed using the Pharmacopoeia of China (China Pharmacopoeia, 2020).The laboratory of Beijing Sifang Chinese medicine pieces Co., Ltd. conducted residue analysis and organic trace analysis (Beijing, China) (C20230506-05) and confirmed that the Impurities, moisture, total ash, sulphur dioxide and pesticide and microbiological contamination was within the guidelines of the Chinese Pharmacopoeia (China Pharmacopoeia, 2020).

Myrrh was obtained from Beijing Sifang Chinese medicine pieces Co., Ltd.(batch number 24052302, Kenya).The identity and purity of the compound were confirmed using the Pharmacopoeia of China (China Pharmacopoeia, 2020).The laboratory of Beijing Sifang Chinese medicine pieces Co., Ltd. conducted residue analysis and organic trace analysis (Beijing, China) (C20240520-01) and confirmed that the Impurities, moisture, total ash, sulphur dioxide and pesticide and microbiological contamination was within the guidelines of the Chinese Pharmacopoeia (China Pharmacopoeia, 2020).

Angelica sinensis was obtained from Beijing Shengshilong Pharmaceutical Co., Ltd.(batch number 2206081, Gansu, China).The identity and purity of the compound were confirmed using the Pharmacopoeia of China (China Pharmacopoeia, 2020).The laboratory of Beijing Shengshilong Pharmaceutical Co., Ltd. conducted residue analysis and organic trace analysis (Beijing, China) (C20220626-12) and confirmed that the Impurities, moisture, total ash, sulphur dioxide contamination was within the guidelines of the Chinese Pharmacopoeia (China Pharmacopoeia, 2020).

Sichuan Pepper was obtained from Beijing Jinchongguang Pharmaceutical Co., Ltd. (batch number 2406110, Sichuan, China). The identity and purity of the compound were confirmed using the Pharmacopoeia of China (China Pharmacopoeia, 2020). The laboratory of Beijing Jinchongguang Pharmaceutical Co., Ltd. conducted residue analysis and organic trace analysis (Beijing, China) (C202406110) and confirmed that the sulphur dioxide, pesticide and microbiological contamination was within the guidelines of the Pharmacopoeia of China (China Pharmacopoeia, 2020).

Chuanxiong was obtained from Beijing Jinchongguang Pharmaceutical Co., Ltd. (batch number 2407033, Sichuan, China). The identity and purity of the compound were confirmed using the Pharmacopoeia of China (China Pharmacopoeia, 2020). The laboratory of Beijing Jinchongguang Pharmaceutical Co., Ltd. conducted residue analysis and organic trace analysis (Beijing, China) (C2024070330) and confirmed that the Impurities, moisture, total ash, sulphur dioxide contamination was within the guidelines of the Chinese Pharmacopoeia (China Pharmacopoeia, 2020).

Safflower was obtained from Beijing Sifang Chinese medicine pieces Co., Ltd.(batch number 24083001, Xinjiang, China).The identity and purity of the compound were confirmed using the Pharmacopoeia of China (China Pharmacopoeia, 2020).The laboratory of Beijing Sifang Chinese medicine pieces Co., Ltd. conducted residue analysis and organic trace analysis (Beijing, China) (C20240520-01) and confirmed that the Impurities, moisture, total ash, sulphur dioxide, pesticide and microbiological contamination was within the guidelines of the Chinese Pharmacopoeia (China Pharmacopoeia, 2020).

Clematis chinensis Osbeck was obtained from Beijing Sifang Chinese medicine pieces Co., Ltd.(batch number 24092301, Anhui, China).The identity and purity of the compound were confirmed using the Pharmacopoeia of China (China Pharmacopoeia, 2020).The laboratory of Beijing Sifang Chinese medicine pieces Co., Ltd. conducted residue analysis and organic trace analysis (Beijing, China) (C20240927-03) and confirmed that the Impurities, moisture, total ash, sulphur dioxide, pesticide and microbiological contamination was within the guidelines of the Chinese Pharmacopoeia (China Pharmacopoeia, 2020).

Dahurian Angelica was obtained from Beijing Jinchongguang Pharmaceutical Co., Ltd. (batch number 2407010, Sichuan, China). The identity and purity of the compound were confirmed using the Pharmacopoeia of China (China Pharmacopoeia, 2020). The laboratory of Beijing Jinchongguang Pharmaceutical Co., Ltd. conducted residue analysis and organic trace analysis (Beijing, China) (C202407010) and confirmed that the Impurities, moisture, total ash, sulphur dioxide contamination was within the guidelines of the Chinese Pharmacopoeia (China Pharmacopoeia, 2020).

FangFeng was obtained from Beijing Shengshilong Pharmaceutical Co., Ltd.(batch number 2406108, Heilongjiang, China).The identity and purity of the compound were confirmed using the Pharmacopoeia of China (China Pharmacopoeia, 2020) and National Standard for the Preparation of Chinese Medicinal Pieces.The laboratory of Beijing Shengshilong Pharmaceutical Co., Ltd. conducted residue analysis and organic trace analysis (Beijing, China) (C20240702-7) and confirmed that the Impurities, moisture, total ash, sulphur dioxide contamination was within the guidelines of the Chinese Pharmacopoeia (China Pharmacopoeia, 2020).

Licorice Root was obtained from Beijing Shengshilong Pharmaceutical Co., Ltd.(batch number 2409036, Nei Mongol Autonomous Region, China).The identity and purity of the compound were confirmed using the Pharmacopoeia of China (China Pharmacopoeia, 2020).The laboratory of Beijing Shengshilong Pharmaceutical Co., Ltd. conducted residue analysis and organic trace analysis (Beijing, China) (20240923-4) and confirmed that the Impurities, moisture, total ash, the heavy metal，harmful element and sulphur dioxide contamination was within the guidelines of the Chinese Pharmacopoeia (China Pharmacopoeia, 2020).

# SECTION 2

The chemical constituents of HTPGP and the transdermally absorbed components after administration were analyzed and identified using ultra-high-performance liquid chromatography coupled with quadrupole–Orbitrap high-resolution mass spectrometry (UHPLC-Q-Exactive Orbitrap HRMS). The specific transdermal components are shown in **Supplementary Table 1**, and the identified herbal chemical constituents of HTPGP are presented in **Supplementary Table 2**.

**Supplementary Table 1.** Chemical constituents detected in tissues after transdermal absorption of HTPGP.

| **NO.** | **tR/s** | **Discriminant** | **Experimental m/z** | **Theoretical m/z** | **Inaccuracies** | **Chemical formula** | **Compound Name** | **MS/MS Fragments** | **Source** | **Class** |
| --- | --- | --- | --- | --- | --- | --- | --- | --- | --- | --- |
| 1* | 351.2 | [M+H]+ | 217.0496 | 217.049559 | 0.2 | C_12_H_8_O_4_ | Methoxsalen | 217.0497894;202.0263672;173.0600128;203.0298309; | DG,BZ,FF，HTP | Coumarins |
| 2* | 417.7 | [M+H]+ | 259.0963 | 259.096509 | 0.8 | C_15_H_14_O_4_ | Xanthoxyletin | 259.0968018;227.0709839;229.0500946;244.0735016;213.0550079;258.1877441;229.085495;228.0762024; | HTP | Coumarins |
| 3 | 267.9 | [M+H]+ | 469.1704 | 469.1704619 | 0.1 | C_22_H_28_O_11_ | Cimifugin glycoside isomers | 469.1709595;307.1184387;184.0737;86.09653473;261.1125488;84.08085632;52.13108063;104.1073608;70.06531525;290.1152954;137.0465546;308.1228943;72.08090973; | FF | Chromanes |
| 4 | 292.6 | [M+H]+ | 307.1175 | 307.1176 | 0.2 | C_16_H_18_O_6_ | Cimifugin | 307.1179504;235.0605469;289.107605;259.0603943;76.0216217;137.0463104;162.0224304; | FF | Chromanes |
| 5 | 298.6 | [M+H]+ | 453.1754 | 453.1755 | 0.3 | C_22_H_28_O_10_ | 4'-O-.beta.-D-Glucosyl-5-O-methylvisamminol | 291.1232605;453.1762085;273.1127625;231.0656433;292.1270447;184.0735931;274.1163025;245.1177368;219.0655518;313.1646118;50.35384369;203.0706329;243.0643463;132.0764771; | FF | Chromanes |
| 6 | 284 | [M-H]- | 417.1186 | 417.1190824 | 1.2 | C_21_H_22_O_9_ | Glycyrrhizin isomers | 255.0658569;135.0085449;119.0501022;256.0688782;417.1184082;153.0190735;136.0113983;91.01896667; | GC | Flavonoids |
| 7 | 467 | [M-H]- | 487.3422 | 487.3428746 | 1.3 | C_30_H_48_O_5_ | Bayogenin | 487.3418274;54.14961243;486.3309021;443.3514709;469.3316956;54.26106262;427.323822; | GC | Triterpenoids |
| 8 | 450.8 | [M-H]- | 487.3422 | 487.3428748 | 1.4 | C_30_H_48_O_5_ | Bayogenin isomers | 487.3418274;54.14961243;486.3309021;443.3514709;469.3316956;54.26106262;427.323822; | GC | Triterpenoids |
| 9 | 450.8 | [M-H]- | 487.3422 | 487.3428748 | 1.4 | C_30_H_48_O_5_ | Belamcanda saponin aglycone analogues | 487.3418274;54.14961243;486.3309021;443.3514709;469.3316956;54.26106262;427.323822; | GC | Triterpenoids |
| 10 | 450.8 | [M-H]- | 487.3422 | 487.342875 | 1.4 | C_30_H_48_O_5_ | Euscaphic acid | 487.3418274;54.14961243;486.3309021;443.3514709;469.3316956;54.26106262;427.323822; | GC | Triterpenoids |
| 11 | 375.8 | [M+H]+ | 260.0918 | 260.0917579 | 0.2 | C_14_H_13_NO_4_ | Skimmianine | 260.0921326;227.0580902;245.0687256;216.0657501;228.0619812;246.0718536;244.0605011; | HTP、HJ | Tryptophan alkaloids |
| 12* | 317.3 | [M+H]+ | 305.1022 | 305.1019883 | 0.6 | C_16_H_16_O_6_ | 9-(2,3-dihydroxy-3-methyl-butoxy)furo[3,2-g]chromen-7-one | 203.0343628;305.102478;204.037674;59.04928207;57.0699501;85.06480408;159.0443115; | HTP,HJ,BZ | Coumarins |
| 13 | 290 | [M-H]- | 191.0348 | 191.034959 | 1 | C_10_H_8_O_4_ | Isoscopoletin | 176.0112;191.0348663;111.0086899;87.00880432;74.02458191;85.02925873;177.014801;120.0214844;91.05548859;147.0451508; | TXTGC | Coumarins |
| 14 | 425.2 | [M+H]+ | 242.1178 | 242.1175788 | 0.8 | C_15_H_15_NO_2_ | N-Methylflindersine | 242.1179504;200.0714417;224.1077118;188.070343;200.1074371;214.1230316;241.1955719; | HTP,HJ | Tryptophan alkaloids |

Notes：HTP：Z.ailanthoides Bark, TXTGC：Clematis intricata Bunge, RX：Frankincense, MY：Myrrh, DG：Angelica sinensis, HJ：Sichuan Pepper, CX：Chuanxiong, HH：Safflower, WLX：Clematis chinensis Osbeck, BZ：Dahurian Angelica, GC：Licorice Root, FF：Fang Feng.All substances included in this table were confirmed by matching with authentic standards in the reference library.

**Supplementary Table 2.** List of herbal constituents identified in HTPGP.

| **NO.** | **tR/s** | **Discriminant** | **Experimental m/z** | **Theoretical m/z** | **Inaccuracies** | **Chemical formula** | **Compound Name** | **MS/MS Fragments** | **Source** | **Class** |
| --- | --- | --- | --- | --- | --- | --- | --- | --- | --- | --- |
| 1 | 213.6 | NEG | 153.0192 | 153.019309 | 0.9 | C_7_H_6_O_4_ | 3,4-Dihydroxybenzoic acid | 109.029335;153.0191345;123.0449982;108.0217438;110.0326691;135.0449829;81.03445435; | CX,HH | Phenolic acids (C6-C1) |
| 2 | 224.2 | NEG | 149.0242 | 149.024359 | 1.3 | C_8_H_8_O_4_ | Vanillic acid | 149.0242004;147.008606;148.0165405;80.05046082;79.95727539;121.029335;77.03958893;105.0343704;139.039978;93.03445435;87.00850677; | CX,HH,WLX,FF | Phenolic acids (C6-C1) |
| 3 | 289.4 | POS | 137.1074 | 137.107348 | 0 | C_8_H_12_N_2_ | Ligustrazine | 137.1074982;43.01794052;95.08564758;109.1013107;81.06998444;93.07001495;109.0650177; | CX,WLX | Tetramate alkaloids |
| 4 | 369.7 | NEG | 119.05 | 119.050215 | 1.5 | C_8_H_8_O | 4-Vinylphenol | 119.0500259; | DG | Phenolic acids (C6-C1) |
| 5 | 489.3 | NEG | 297.1491 | 297.1495947 | 1.7 | C_19_H_22_O_3_ | 6-[(2E)-3,7-dimethylocta-2,6-dienyl]-7-hydroxy-chromen-2-one | 296.230896;278.220459;297.1487122;196.1421661;195.1389313;171.1024017;172.1060638;279.2236938;197.1446381;174.0317535;59.0137825;184.1064453;173.1087189;228.0799561;183.1031952;277.2165833; | DG | Coumarins |
| 6 | 293.2 | NEG | 367.1028 | 367.1034324 | 1.6 | C_17_H_20_O_9_ | Cnidioside A | 205.0503082;161.0606232;191.0559235;173.0453033;193.0503845;206.0538483;187.0398407;93.03447723;162.0641479;367.103363;134.0370483; | DG | Coumarins |
| 7 | 305.2 | NEG | 151.0398 | 151.040044 | 1.3 | C_8_H_8_O_3_ | 2',4'-Dihydroxyacetophenone | 151.0399017;150.0557709;108.0215149;136.0164337;107.0501022;109.0296021;93.02197266;123.0448685;121.0168839;124.0399399;94.03020477;59.01382828;81.03452301;65.03959656;137.0199585; | DG | Shikimates and Phenylpropanoids |
| 8 | 328.8 | NEG | 135.045 | 135.04513 | 1 | C_8_H_8_O_2_ | Phenylacetic acid | 135.0449219;134.0371399;107.0501709; | DG | Shikimates and Phenylpropanoids |
| 9 | 394.5 | NEG | 279.1597 | 279.1601592 | 1.7 | C_16_H_24_O_4_ | Kaempferol-3-O-diglucosylrutinoside | 279.159668;235.1698761;251.164917;59.01378632;278.1403198;57.03451538;217.1594849;191.1435394;85.02935028;235.1340179;125.0969086;237.1493073; | DG | Macrolides |
| 10 | 333.6 | POS | 287.0916 | 287.0913883 | 0.8 | C_16_H_16_O_6_ | 4-(2,3-dihydroxy-3-methyl-butoxy)furo[3,2-g]chromen-7-one | 286.2169189;258.1856689;121.0285873;259.1892395;287.0922241;287.054657;270.1498718;245.0813293;193.0494995;203.0343781;107.0493469; | DG,BZ | Coumarins |
| 11 | 422.6 | POS | 231.1017 | 231.101594 | 0.6 | C_14_H_14_O_3_ | Demethylsuberosin | 231.1018066;175.0391083;176.0432434;230.1542206;119.0856094;173.1324463;185.1326904;213.1278076;145.1014099;171.1168365;157.1011047; | DG,BZ | Coumarins |
| 12 | 241.2 | NEG | 353.0869 | 353.087782 | 2.5 | C_16_H_18_O_9_ | Scopolin | 173.0452423;179.0346985;191.0558624;135.0449677;353.0872192;155.0348053;93.03446198;180.0383453;174.0485687;137.0244141; | DG,BZ | Coumarins |
| 13 | 295.7 | NEG | 193.0504 | 193.050609 | 1.2 | C_10_H_10_O_4_ | Methyl caffeate | 134.0370789;178.0267944;193.0502625;149.0605774;135.04039; | DG,BZ | Phenylpropanoids (C6-C3) |
| 14 | 332.4 | POS | 335.1127 | 335.112553 | 0.4 | C_17_H_18_O_7_ | Byakangelicin | 231.0292358;233.0448608;335.1129456;232.0326996;85.06489563;234.0480804;317.102417;67.05432892;299.0918579;149.0965424;317.2153015;218.0216675;299.2012939;334.2411194;159.1173401;95.08605194; | DG,BZ,FF | Coumarins |
| 15 | 319.6 | NEG | 201.0191 | 201.019309 | 1.2 | C_11_H_6_O_4_ | Xanthoxyletin | 201.0190125;173.0241089;157.0292358;200.1287994;116.0715027;145.0293579;111.0814056;117.0344009;59.01377869;156.1388245;147.0446777; | DG,BZ,FF | Coumarins |
| 16 | 289.8 | POS | 409.1498 | 409.149332 | 1.1 | C_20_H_24_O_9_ | Nodakenin | 247.0970612;229.0862579;246.1489716;408.1981201;187.0391083;70.06522369;248.1005249;228.1380768;85.02864075;112.0871048;248.1294861; | DG,BZ,FF | Coumarins |
| 17 | 433.9 | POS | 191.1067 | 191.1066797 | 0.1 | C_12_H_14_O_2_ | Ligustilide | 191.106842;173.0962982;145.1013336;163.1119537;91.05435181;149.0599365;117.0700455;155.0858154;105.0699463;93.06990814;79.05428314; | DG,CX | Cyclic polyketides |
| 18 | 413.4 | POS | 191.1067 | 191.1066797 | 0.2 | C_12_H_14_O_2_ | Butylphthalide | 145.1013336;173.096344;191.107132;135.04422;91.05430603;131.0493469;146.1047668;117.0699768;135.1170959;105.0700684;131.0856628; | DG,CX | Cyclic polyketides |
| 19 | 408.7 | NEG | 203.071 | 203.0713443 | 1.5 | C_12_H_12_O_3_ | (3Z)-3-butylidene-5-hydroxy-isobenzofuran-1-one | 203.0710449;160.0163422;174.0319214;161.0198212;161.0969849;173.0240631;175.0355377;144.0579224; | DG,CX | Cyclic polyketides |
| 20 | 352.4 | POS | 187.039 | 187.038994 | 0.1 | C_11_H_6_O_3_ | Psoralen | 187.0391235;143.0493622;131.0492706;115.0543137; | DG,FF | Chromanes |
| 21 | 407.4 | POS | 229.086 | 229.0859442 | 0.3 | C_14_H_12_O_3_ | Xanyhyletin | 229.0846558;228.102356;186.0546722;214.0625;174.0549316;210.092041;211.0750427; | DG,HJ | Coumarins |
| 22 | 296.6 | POS | 163.039 | 163.038994 | 0.1 | C_9_H_6_O_3_ | Umbelliferone | 163.0391235;119.0492706;107.049263;91.05435181;107.0856018;145.1013489;119.0856094;93.0699234;43.01795197;137.0962524;135.1170502; | DG,HJ,BZ,GC | Coumarins |
| 23 | 255 | NEG | 93.0344 | 93.034565 | 1.4 | C_6_H_6_O | Phenol | 93.03445435; | DG,WLX | 莽草酸及苯丙酸类 |
| 24 | 383.1 | POS | 277.1072 | 277.1070737 | 0.5 | C_15_H_16_O_5_ | Hamaudol | 277.1073914;259.0968933;205.0498047;149.0598907;276.2015381;86.06014252;206.1542053;191.1069641;260.1000671;276.2805176;121.1012268;133.1012421;107.0857315;69.06999207;105.0699463;217.0500031; | FF | Chromanes |
| 25 | 44.6 | NEG | 88.0403 | 88.040379 | 1 | C_3_H_7_NO_2_ | Alanine | 88.04032898;59.01380539;71.01377869;87.0087204;41.00331879;43.01897812;44.99819946; | GC | Small peptides |
| 26 | 434.9 | POS | 383.1491 | 383.1489386 | 0.4 | C_22_H_22_O_6_ | Licoricone | 327.086853;328.09021;299.0918274;178.0628967;312.0630188;191.0710449;383.1551819;295.0601807;191.1063232;147.1169739;95.08554077; | GC | Isoflavonoids |
| 27 | 413.3 | POS | 353.1021 | 353.1019883 | 0.4 | C_20_H_16_O_6_ | Semilicoisoflavone B | 353.102417;153.0185242;311.0552063;335.0921631;183.1014862;317.2111816;307.0975647;190.0867004;335.2221985;227.0708618;95.08566284; | GC | Isoflavonoids |
| 28 | 504.3 | NEG | 469.3316 | 469.33231 | 1.5 | C_30_H_46_O_4_ | 18α-Glycyrrhetinic acid | 469.3313293;52.14842606;383.2962036;425.3424072;468.3191833;373.2738953;409.3111572;52.25993729; | GC | Triterpenoids |
| 29 | 340.5 | POS | 257.0808 | 257.0808589 | 0.1 | C_15_H_12_O_4_ | Glycyrrhizin | 257.0812378;137.0235138;147.0442657;71.04924774;185.1326904;239.0707245;211.075882;242.057785;163.0392761;157.1015778;138.0271149; | GC | Flavonoids |
| 30 | 262.1 | POS | 595.1663 | 595.1657705 | 0.9 | C_27_H_30_O_15_ | 5,7-dihydroxy-2-(4-hydroxyphenyl)-6,8-bis[3,4,5-trihydroxy-6-(hydroxymethyl)tetrahydropyran-2-yl]chromen-4-one | 457.1134033;325.0713196;379.0816956;409.0924683;337.0710754;427.102478;439.1029968;421.092865;481.1133423;391.0817566;541.1350098; | GC | Flavonoids |
| 31 | 45 | NEG | 146.0457 | 146.045858 | 1 | C_5_H_9_NO_4_ | Glutamate | 102.0559158;128.0351715;146.0458374;127.0511627;145.0617828;109.040657;74.02465057;41.99851227;84.0453949; | GC | Small peptides |
| 32 | 289.3 | POS | 551.1763 | 551.1759412 | 0.7 | C_26_H_30_O_13_ | 2-[4-[3-[3,4-dihydroxy-4-(hydroxymethyl)tetrahydrofuran-2-yl]oxy-4,5-dihydroxy-6-(hydroxymethyl)tetrahydropyran-2-yl]oxyphenyl]-7-hydroxy-chroman-4-one | 114.1277924;257.0812988;70.06520081;550.2527466;115.1313019;550.3439331;86.09648132;84.0809021;85.02866364;268.0824585;137.059906; | GC | Flavonoids |
| 33 | 299.1 | NEG | 163.0399 | 163.040044 | 1.1 | C_9_H_8_O_3_ | 4-Hydroxycinnamic acid | 119.0500336;163.039917;120.0534058;162.0276031; | GC | Phenylpropanoids (C6-C3) |
| 34 | 463.9 | NEG | 391.191 | 391.1914595 | 1.1 | C_25_H_28_O_4_ | 7-hydroxy-2-[4-hydroxy-3-(3-methylbut-2-enyl)phenyl]-8-(3-methylbut-2-enyl)chroman-4-one | 187.1125488;203.071106;391.1897888;221.081543;188.1159668;159.0813751;132.0580139;204.0742035;79.95730591;59.01382065;83.04994965;347.2211609;157.0652161;222.0856934;80.96528625; | GC | Flavonoids |
| 35 | 337.8 | POS | 177.0546 | 177.0546088 | 0.2 | C_10_H_10_O_4_ | Dimethyl phthalate | 177.0548096;133.0649414;121.0650024;176.1063385;159.1170959;149.059906;131.0858612;149.0964203;107.0857849;145.0286713;43.01792526;93.07000732;135.1167145;105.0700378;135.0807648; | GC | Phenolic acids (C6-C1) |
| 36 | 279.6 | POS | 565.1559 | 565.1552056 | 1.2 | C_26_H_28_O_14_ | Schaftoside | 409.0922852;427.1028748;379.0817871;511.1235352;391.0817566;325.0711365;529.1337891;349.0713501;337.0711975;481.112915;295.0603333;499.1234741;433.0923767;547.1427612;493.1135864;397.0921021; | GC | Flavonoids |
| 37 | 279.6 | POS | 565.1559 | 565.1552058 | 1.2 | C_26_H_28_O_14_ | 5,7-dihydroxy-2-(4-hydroxyphenyl)-8-[3,4,5-trihydroxy-6-(hydroxymethyl)tetrahydropyran-2-yl]-6-(3,4,5-trihydroxytetrahydropyran-2-yl)chromen-4-one | 409.0922852;427.1028748;379.0817871;511.1235352;391.0817566;325.0711365;529.1337891;349.0713501;337.0711975;481.112915;295.0603333; | GC | Flavonoids |
| 38 | 270.2 | NEG | 563.1395 | 563.1406056 | 2 | C_26_H_28_O_14_ | Vicenin-1 | 563.1392822;353.0660095;383.0765686;443.0977173;473.1080933;173.0452881;354.069458;503.118927;191.055542;384.0804443;413.086731; | GC | Flavonoids |
| 39 | 279.6 | POS | 565.1559 | 565.155206 | 1.2 | C_26_H_28_O_14_ | Vicenin-3 | 409.0922852;427.1028748;379.0817871;511.1235352;391.0817566;325.0711365;529.1337891;349.0713501;337.0711975;481.112915;295.0603333; | GC | Flavonoids |
| 40 | 279.6 | POS | 565.1559 | 565.1552058 | 1.2 | C_26_H_28_O_14_ | 5,7-dihydroxy-2-(4-hydroxyphenyl)-6-[3,4,5-trihydroxy-6-(hydroxymethyl)tetrahydropyran-2-yl]-8-(3,4,5-trihydroxytetrahydropyran-2-yl)chromen-4-one | 409.0922852;427.1028748;379.0817871;511.1235352;391.0817566;325.0711365;529.1337891;349.0713501;337.0711975;481.112915;295.0603333; | GC | Flavonoids |
| 41 | 279.6 | POS | 565.1559 | 565.155206 | 1.2 | C_26_H_28_O_14_ | Isoschaftoside | 409.0922852;427.1028748;379.0817871;511.1235352;391.0817566;325.0711365;529.1337891;349.0713501;337.0711975;481.112915;295.0603333;499.1234741;433.0923767;547.1427612;493.1135864;397.0921021; | GC | Flavonoids |
| 42 | 381.2 | NEG | 255.0659 | 255.0662589 | 1.4 | C_15_H_12_O_4_ | 5,7-dihydroxy-2-phenyl-chroman-4-one | 119.050087;135.0085907;255.065918;153.0191498;91.01883698;120.0534592;136.0119171;211.1338806;254.1478424;210.1579437;149.0242157;213.0547485;87.04524994;59.01382065;57.03448105; | GC | Flavonoids |
| 43 | 285.1 | POS | 579.1715 | 579.170856 | 1.2 | C_27_H_30_O_14_ | Violanthin | 525.1395264;379.0818481;337.0712585;325.071228;423.1083069;405.0973206;543.149292;441.1184692;393.0973206;457.1139526;355.0814514;507.1288147;421.0921631; | GC | Flavonoids |
| 44 | 495.8 | NEG | 821.3958 | 821.3964859 | 0.9 | C_42_H_62_O_16_ | Diammonium Glycyrrhizinate | 821.3950195;351.0561523;113.0242615;193.0350037;91.26722717;175.0245361;99.00871277;352.0597229;759.394165;103.0035019;645.3625488; | GC | Triterpenoids |
| 45 | 495.8 | NEG | 821.3958 | 821.3964859 | 0.9 | C_42_H_62_O_16_ | Glycyrrhizin | 821.3950195;351.0561523;113.0242615;193.0350037;91.26722717;175.0245361;99.00871277;352.0597229;759.394165;103.0035019;645.3625488; | GC | Triterpenoids |
| 46 | 495.8 | NEG | 821.3958 | 821.3964859 | 0.9 | C_42_H_62_O_16_ | Licorice-saponin H2 | 821.3950195;351.0561523;113.0242615;193.0350037;91.26722717;175.0245361;99.00871277;352.0597229;759.394165;103.0035019;645.3625488; | GC | Triterpenoids |
| 47 | 333.6 | POS | 287.0916 | 287.091424 | 0.7 | C_16_H_14_O_5_ | Licochalcone B | 286.2169189;258.1856689;121.0285873;259.1892395;287.0922241;287.054657;270.1498718;245.0813293;193.0494995;203.0343781;107.0493469; | GC | Flavonoids |
| 48 | 381.5 | POS | 257.0811 | 257.080859 | 0.9 | C_15_H_12_O_4_ | Isoliquiritigenin | 257.0812988;137.0235443;147.0443115;256.2062988;239.0708008;256.1346436;211.0759125;145.1016083;199.148468;242.0586395;157.1016083; | HTP,GC | Flavonoids |
| 49 | 320.8 | POS | 431.1337 | 431.133682 | 0.2 | C_22_H_22_O_9_ | Ononin | 269.0810242;270.0845337; | HTP,GC | Isoflavonoids |
| 50 | 52.8 | POS | 144.1019 | 144.101929 | 0.1 | C_7_H_13_NO_2_ | Stachydrine | 144.1019897;84.08084106;98.09649658;70.06522369;85.084198;99.04414368;85.02852631; | HTP,GC | Ornithine alkaloids |
| 51 | 497.8 | NEG | 405.1702 | 405.1707241 | 1.2 | C_25_H_26_O_5_ | 6,8-Diprenylgenistein | 405.1694031;361.2391663;350.1154175;307.0602112;57.03447342;59.01379395;96.96006012;78.95876312;167.0015717; | HTP,GC | Isoflavonoids |
| 52 | 46.9 | NEG | 503.1611 | 503.161735 | 1.2 | C_18_H_32_O_16_ | Raffinose | 89.02416992;179.0555115;383.1175232;101.0243454;59.01376343;71.01378632;119.0349884;113.0243607;221.0654755;161.0453796;503.1611938;143.0346832;73.02929688;103.9107895;318.666748;200.8275757; | HTP,MY | Saccharides |
| 53 | 46.9 | NEG | 503.1611 | 503.161735 | 1.2 | C_18_H_32_O_16_ | Gentianose | 89.02416992;179.0555115;383.1175232;101.0243454;59.01376343;71.01378632;119.0349884;113.0243607;221.0654755;161.0453796;503.1611938; | HTP,MY | Saccharides |
| 54 | 45.6 | POS | 203.0527 | 203.052688 | 0 | C_6_H_12_O_6_ | Allose | 203.052948;143.0193024;161.0296478;101.0081024;60.05568314; | HH | Saccharides |
| 55 | 414.9 | POS | 304.1445 | 304.144462 | 0.3 | C_19_H_17_N_3_O | (±)-Evodiamine | 134.0601501;304.1449585;171.0919342;161.0711212;144.0809479;303.2327271;95.08570862;133.1012421;121.1010895;135.0635529;81.06989288; | HH | Anthranilic acid alkaloids |
| 56 | 316 | POS | 449.1081 | 449.1078616 | 0.6 | C_21_H_20_O_11_ | Kaempferol-7-O-glucoside | 287.0553589;216.1749115;288.0587463;448.2658691;448.3002014;85.02856445;199.0960846;130.0865784;164.0709381;70.0652771;69.03368378;71.04927063; | HH | Flavonoids |
| 57 | 414.9 | POS | 304.1445 | 304.144462 | 0.3 | C_19_H_17_N_3_O | Evodiamine | 134.0601501;304.1449585;171.0919342;161.0711212;144.0809479;303.2327271;95.08570862;133.1012421;121.1010895;135.0635529;81.06989288; | HH | Anthranilic acid alkaloids |
| 58 | 349.1 | POS | 584.2756 | 584.2755361 | 0.1 | C_34_H_37_N_3_O_6_ | (E)-3-(4-hydroxyphenyl)-N-[4-[[(E)-3-(4-hydroxyphenyl)prop-2-enoyl]-[3-[[(E)-3-(4-hydroxyphenyl)prop-2-enoyl]amino]propyl]amino]butyl]prop-2-enamide | 438.2391052;204.1021118;420.2287598;147.04422;292.202301;439.2423706;275.1755371;421.2261047;218.1177521;584.2727051;205.1055756;217.1341553; | HH | Ornithine alkaloids |
| 59 | 378.8 | NEG | 313.1078 | 313.1081238 | 1.1 | C_18_H_18_O_5_ | -(4-hydroxyphenyl)ethyl (E)-3-(4-hydroxy-3-methoxy-phenyl)prop-2-enoate | 193.0503387;134.0371552;59.01377869;149.0605316;179.0345001;313.108429;85.0293808;194.0539093;269.1752319;178.0267487;135.0414581; | HH | Phenylpropanoids (C6-C3) |
| 60 | 52.8 | POS | 110.0601 | 110.060064 | 0.3 | C_6_H_7_NO | 3-Pyridinemethanol | 110.0600357;109.0479813;81.03359985; | HH | Nicotinic acid alkaloids |
| 61 | 45.8 | NEG | 209.0664 | 209.0666528 | 1.2 | C_7_H_14_O_7_ | D-Mannoheptulose | 85.02940369;209.0665131;129.0192108;87.00867462;59.01379013;57.03452682;159.0296783;89.02430725;71.01378632;99.0087204;111.0085602; | HH | Saccharides |
| 62 | 251.9 | NEG | 269.1025 | 269.1030384 | 2.1 | C_13_H_18_O_6_ | (2R,3R,4S,5S,6R)-2-benzyloxy-6-(hydroxymethyl)tetrahydropyran-3,4,5-triol | 59.01377869;269.1029053;101.0242996;71.01374817;73.02941895;85.02938843;113.0242615;225.1132507;57.03447342;133.0138397;83.0137558; | HH | Shikimates and Phenylpropanoids |
| 63 | 331.1 | NEG | 445.0772 | 445.077611 | 0.9 | C_21_H_18_O_11_ | Baicalin | 269.0453491;128.9828644;113.0243683;85.02944946;59.0138092;270.0488281;100.9879456;179.0349579;163.0399475;121.0293961;145.0293579; | HH | Flavonoids |
| 64 | 229.1 | NEG | 109.0293 | 109.029479 | 1.4 | C_6_H_6_O_2_ | Pyrocatechol | 109.0293427;108.0215912; | HH | Shikimates and Phenylpropanoids |
| 65 | 53.8 | POS | 133.0497 | 133.049524 | 1 | C_5_H_10_O_5_ | Xylose | 86.09653473;97.02854156;132.1021271;87.09981537;69.03364563;73.02852631;74.02376556;133.0494385;87.05541992; | HH | Saccharides |
| 66 | 333 | NEG | 151.0399 | 151.040044 | 0.9 | C_8_H_8_O_3_ | Methyl Paraben | 151.0398865;93.03451538;107.050148;136.016449;92.02666473;77.03948212; | HH | Phenolic acids (C6-C1) |
| 67 | 50.5 | POS | 325.1133 | 325.112912 | 1.2 | C_12_H_22_O_11_ | Isomaltose | 85.02843475;127.0390625;145.0498657;174.9701233;91.03897858;186.9682159;97.02844238;232.862442;69.03335571;96.37595367;55.89219284;51.73369217;252.8217163;241.7952118;112.9525528;52.8841362;63.84457397;79.78144073; | HH | Saccharides |
| 68 | 153.7 | NEG | 164.0715 | 164.071679 | 1.2 | C_9_H_11_NO_2_ | D-Phenylalanine | 120.0453568;94.02971649;147.0449524;164.0715179;72.00901794;164.0352173;121.0293732;66.0348587;121.0495758;92.05041504;91.05517578; | HH | Small peptides |
| 69 | 49.6 | NEG | 131.0348 | 131.034924 | 0.9 | C_5_H_10_O_5_ | Ribose | 57.03451538;131.0347748;87.04502106;69.03451538;85.02939606;113.0243454;59.01377487;58.00596237;73.02940369;59.0501709;88.04035187; | HH | Saccharides |
| 70 | 265.8 | NEG | 611.1613 | 611.161735 | 0.8 | C_27_H_32_O_16_ | Hydroxysafflor yellow A | 611.1608887;491.1186829;325.0712891;403.1027832;473.1080322;283.0611877;295.0606995;313.0714111;492.1217346;207.0508881;119.050087; | HH | Flavonoids |
| 71 | 591.4 | POS | 338.3422 | 338.341765 | 1.4 | C_22_H_43_NO | Erucamide | 338.3421021;83.08561707;97.10121918;69.06996155;57.06996155;321.3156433;71.08561707;95.08561707;81.07001495;303.3051453;111.1169891; | HH | Fatty amides |
| 72 | 298.9 | NEG | 177.0191 | 177.019309 | 1.4 | C_9_H_6_O_4_ | 5,7-Dihydroxychromone | 133.0292664;134.0371399;177.019516;105.0344086;93.03440094;147.0448914;135.0449524;119.0498886;77.03961945;95.05012512;131.0498962; | HH | Chromanes |
| 73 | 222.9 | POS | 355.1027 | 355.102382 | 0.9 | C_16_H_18_O_9_ | Chlorogenic acid | 192.1019135;206.0813446;188.0709534;338.1395264;163.0391541;190.0865173; | HH | Phenylpropanoids (C6-C3) |
| 74 | 352.8 | NEG | 285.0402 | 285.040438 | 0.7 | C_15_H_10_O_6_ | Luteolin | 285.0400696;284.1580811;59.0137558;241.1442108;255.0658722;151.0033875;87.04502869;73.02947235;197.1543884;57.03450775; | HH | Flavonoids |
| 75 | 372.8 | NEG | 269.0452 | 269.0454882 | 1 | C_15_H_12_O_6_ | Eriodictyol | 269.0453186;225.1494141;268.1629639;87.0450058;251.1648865;59.01376724;181.1596222;99.00868988;57.03450012;207.1391296; | HH | Flavonoids |
| 76 | 277.2 | POS | 449.1085 | 449.1078615 | 1.4 | C_21_H_20_O_11_ | Isoorientin | 329.0660706;299.0554199;353.06604;287.0553894;395.0767822;413.0871277;383.076416;431.0973511;365.0660706;339.0871582;377.0657654; | HH,GC | Flavonoids |
| 77 | 303.8 | POS | 625.1767 | 625.176335 | 0.6 | C_28_H_32_O_16_ | Narcissin | 317.0658569;318.069397;85.02848816;71.04922485;302.0423889;129.0547638;479.1191406;624.3521118;70.06523895; | HH,GC | Flavonoids |
| 78 | 309.6 | POS | 595.1661 | 595.1657703 | 0.5 | C_27_H_30_O_15_ | Nicotiflorin | 287.0552979;85.02848053;288.0587158;71.04920959;129.0548401;594.3434448;70.06533813;433.1138;301.0704651;97.02887726;145.0500031;127.0391617;453.1786804; | HH,GC | Flavonoids |
| 79 | 284.2 | POS | 139.1118 | 139.111765 | 0.4 | C_9_H_14_O | 3,5,5-Trimethyl-2-cyclohexen-1-one | 139.1118317;43.01792526;95.08560181;121.1013184;93.06999207;81.06995392;97.06495667;45.03358078;111.0804977;57.03361511;96.08883667;71.04915619; | HH,GC | Apocarotenoids |
| 80 | 50.5 | POS | 150.0914 | 150.091329 | 0.3 | C_9_H_13_NO_2_ | Synephrine | 150.0912781;135.0679169;119.0491562;91.05423737;121.0648575;109.0648041;132.0808258; | HH,WLX | Tyrosine alkaloids |
| 81 | 105.5 | POS | 127.0389 | 127.038994 | 0.6 | C_6_H_6_O_3_ | 5-Hydroxymethylfurfural | 127.0394211;109.0285721;108.0445557;69.03362274;126.0551682;43.01795959;81.03364563;99.04419708;81.07002258;55.01798248;53.03870773； | HH,WLX | Cyclic polyketides |
| 82 | 337.8 | POS | 177.0546 | 177.054644 | 0 | C_10_H_8_O_3_ | 7-Methoxycoumarin | 177.0548096;133.0649414;121.0650024;176.1063385;159.1170959;149.059906;131.0858612;149.0964203;107.0857849;145.0286713;43.01792526; | HJ | Coumarins |
| 83 | 302.2 | POS | 609.1817 | 609.18142 | 0.5 | C_28_H_32_O_15_ | Diosmin | 301.0709534;463.1242065;302.0745239;464.1277466;85.02848816;71.04932404 | HJ | Flavonoids |
| 84 | 280 | POS | 340.1548 | 340.154358 | 1.3 | C_20_H_21_NO_4_ | Canadine | 340.1546021;176.070816;178.0500793;149.0599518;177.074234;165.0912628;174.055069;144.0808105;136.0621796;179.0534363; | HJ | Tyrosine alkaloids |
| 85 | 360.8 | NEG | 361.1653 | 361.1656387 | 1 | C_20_H_26_O_6_ | Bis(guaiacyl)butanediol | 361.1644897;346.1417236;287.0925598;59.01381302;243.0661926;347.1448975;317.1748352;171.1024628;57.03456879;244.0735779; | HJ | Lignans |
| 86 | 448.5 | POS | 245.1174 | 245.117244 | 0.7 | C_15_H_16_O_3_ | Suberosin | 244.1698151;245.118042;69.06987762 | HJ,BZ | Coumarins |
| 87 | 298.6 | POS | 611.197 | 611.1970706 | 0.1 | C_28_H_34_O_15_ | Hesperetin 7-O-neohesperidoside | 303.0874939;303.0494385;85.02853394;71.04924011;195.0292206;177.0548859;153.0182343;263.0559387;129.0549164;304.091217;413.1242676; | HJ,WLX | Flavonoids |
| 88 | 298.6 | POS | 611.197 | 611.19707 | 0.1 | C_28_H_34_O_15_ | Hesperidin | 303.0874939;303.0494385;85.02853394;71.04924011;195.0292206;177.0548859;153.0182343;263.0559387;129.0549164;304.091217; | HJ,WLX | Flavonoids |
| 89 | 151.2 | POS | 289.0922 | 289.0918176 | 1.2 | C_12_H_16_O_8_ | Maltol-3-O-glucoside | 127.0392151;271.2426758;149.132782;123.117012;81.07003784;109.1014023;95.08575439;121.1014023;135.1171112;107.0857468; | MY | Cyclic polyketides |
| 90 | 323.9 | NEG | 477.0672 | 477.067441 | 0.5 | C_21_H_18_O_13_ | Miquelianin | 301.0349426;302.0384827;151.00354;178.9984283;477.06427;59.01381683;113.0243607;71.01382446;283.0248718;273.0397644;121.0294571;85.02950287;255.0299683;163.0035095; | MY | Flavonoids |
| 91 | 71.3 | NEG | 173.0089 | 173.009103 | 1.1 | C_6_H_8_O_7_ | Isocitric acid | 111.0086365;85.02940369;59.01379013;129.0555573;129.0191803;154.9985199;57.03453827;128.0713348;155.034668;173.0089417; | MY | Fatty Acids and Conjugates |
| 92 | 207.4 | NEG | 94.0297 | 94.02981378 | 1.2 | C_5_H_5_NO | Pyrrole-2-carboxaldehyde | 94.02970886;66.03482056;93.03456879; | MY | Proline alkaloids |
| 93 | 132.1 | NEG | 149.0089 | 149.009138 | 1.4 | C_4_H_6_O_6_ | Tartaric acid | 149.0092316;87.00865936;72.9930191;59.01377869;89.02430725;103.0035553;121.0293427;139.0397339;43.01893234;75.00878143;105.0193024; | MY | Fatty Acids and Conjugates |
| 94 | 70.7 | POS | 124.0394 | 124.039328 | 0.5 | C_6_H_5_NO_2_ | Nicotinate | 124.0393906;119.0191269;101.0084152;80.04956818;100.0244904;96.04462433; | MY,DG | Nicotinic acid alkaloids |
| 95 | 75.5 | NEG | 243.0621 | 243.062236 | 0.7 | C_9_H_12_N_2_O_6_ | Uridine | 110.0246201;200.0561981;128.0351563;243.0626678;152.0350189;82.0297699;180.0662079;242.066803;224.0561981;140.0351105;84.04536438; | MY,DG | Nucleosides |
| 96 | 435 | POS | 195.138 | 195.13798 | 0.2 | C_12_H_18_O_2_ | Sedanolide | 149.1326599;125.0598221;79.05431366;177.1276093;195.138092;97.06488037;81.06999207;93.06996918;111.0441589;107.0856476;135.1170044; | MY,DG,CX,FF | Cyclic polyketides |
| 97 | 76.2 | POS | 136.0619 | 136.061795 | 0.8 | C_5_H_5_N_5_ | Adenine | 136.0618591; | MY,DG,CX,HH | Pseudoalkaloids |
| 98 | 281.1 | POS | 153.0547 | 153.054644 | 0.5 | C_8_H_8_O_3_ | Vanillin | 125.0597992;153.0548706;93.03356171;111.0441742;107.0856628;43.01793671;111.0805664;135.0805664;65.03869629;109.0649414;95.04920959; | MY,DG,CX,WLX | Phenolic acids (C6-C1) |
| 99 | 45.6 | POS | 203.0527 | 203.052688 | 0 | C_6_H_12_O_6_ | Galactose | 203.052948;143.0193024;161.0296478;101.0081024;60.05568314; | MY,DG,GC | Saccharides |
| 100 | 45.6 | POS | 203.0527 | 203.052688 | 0 | C_6_H_12_O_6_ | Glucose | 203.052948;143.0193024;161.0296478;101.0081024;60.05568314; | MY,DG,GC | Saccharides |
| 101 | 307.1 | NEG | 161.0453 | 161.045488 | 1.2 | C_6_H_12_O_6_ | D-(+)-Mannose | 160.8418732;59.01377106;85.02935028;161.045517;131.034729;71.01383209;89.02422333;73.0295105;83.01369476;81.0344696;87.00879669; | MY,DG,GC | Saccharides |
| 102 | 104.9 | NEG | 282.0841 | 282.084369 | 0.8 | C_10_H_13_N_5_O_5_ | Guanosine | 150.0419617;282.0834045;59.01379776;133.0154877;151.0457306;94.02970886;281.2480164;108.0202789;238.1208038; | MY,DG,HH | Nucleosides |
| 103 | 53.8 | POS | 133.0497 | 133.049524 | 1 | C_5_H_10_O_5_ | Arabinose | 86.09653473;97.02854156;132.1021271;87.09981537;69.03364563;73.02852631;74.02376556;133.0494385;87.05541992; | MY,HH | Saccharides |
| 104 | 103.2 | NEG | 131.0349 | 131.034924 | 0.3 | C_5_H_10_O_5_ | Lyxose | 87.04502869;131.0348358;57.03451157;85.02936554;130.0872192;73.02942657;113.024292;69.03455353;59.01377106; | MY,HH | Saccharides |
| 105 | 316 | POS | 449.1081 | 449.1078616 | 0.6 | C_21_H_20_O_11_ | Kaempferol-3-O-galactoside | 287.0553589;216.1749115;288.0587463;448.2658691;448.3002014;85.02856445;199.0960846;130.0865784;164.0709381; | MY,HH,GC | Flavonoids |
| 106 | 611.1 | NEG | 455.3523 | 455.353045 | 1.7 | C_30_H_48_O_3_ | β-Boswellic acid | 455.3522339;50.59518051;377.3190613;50.70671082; | RX | Triterpenoids |
| 107 | 316.9 | NEG | 191.0347 | 191.034959 | 1.2 | C_10_H_8_O_4_ | 4-Methylesculetin | 85.02940369;176.0113068;191.0349884;57.03452301;176.0470734;59.01379395;111.0086441;147.0811157;135.0813446; | RX | Coumarins |
| 108 | 50.5 | POS | 325.1133 | 325.112912 | 1.2 | C_12_H_22_O_11_ | D-(+)-Cellobiose | 85.02843475;127.0390625;145.0498657;174.9701233;91.03897858;186.9682159;97.02844238;232.862442;69.03335571; | RX,MY | Saccharides |
| 109 | 50.5 | POS | 325.1133 | 325.112912 | 1.2 | C_12_H_22_O_11_ | Cellobiose | 85.02843475;127.0390625;145.0498657;174.9701233;91.03897858;186.9682159;97.02844238;232.862442;69.03335571; | RX,MY | Saccharides |
| 110 | 267.8 | NEG | 179.0347 | 179.034959 | 1.4 | C_9_H_8_O_4_ | Caffeic acid | 135.0449829;179.0347137;136.0483856; | TXTGC,CX,HH | Phenylpropanoids (C6-C3) |
| 111 | 302.2 | POS | 177.0547 | 177.0546088 | 0.4 | C_10_H_10_O_4_ | Ferulate | 177.0549774;145.0286102;149.0599365;159.1171112;117.0337143;163.0392303;131.0857544;107.0856323;149.0962372; | TXTGC,DG,HH,BZ | Phenylpropanoids (C6-C3) |
| 112 | 295.8 | POS | 611.1614 | 611.160685 | 1.2 | C_27_H_30_O_16_ | Rutin | 303.050354;85.02850342;304.0534058;71.04927063;129.054657;287.055542;70.06499481;610.3270874;317.0663147; | TXTGC,DG,HH,GC | Flavonoids |
| 113 | 372.8 | NEG | 269.0452 | 269.045523 | 1.1 | C_15_H_10_O_5_ | Apigenin | 269.0453186;225.1494141;268.1629639;87.0450058;251.1648865;59.01376724;181.1596222;99.00868988;57.03450012; | TXTGC,HH | Flavonoids |
| 114 | 44.6 | NEG | 181.0715 | 181.071738 | 1.2 | C_6_H_14_O_6_ | Galactitol | 181.0716858;89.02428436;59.01376724;101.0242691;71.01376343;73.02940369;85.02941132;163.0617065;119.0348282; | TXTGC,HH,FF | Saccharides |
| 115 | 44.6 | NEG | 181.0715 | 181.071738 | 1.2 | C_6_H_14_O_6_ | Glucitol | 181.0716858;89.02428436;59.01376724;101.0242691;71.01376343;73.02940369;85.02941132;163.0617065;119.0348282; | TXTGC,HH,FF | Saccharides |
| 116 | 44.6 | NEG | 181.0715 | 181.071738 | 1.2 | C6H14O6 | Mannitol | 181.0716858;89.02428436;59.01376724;101.0242691;71.01376343;73.02940369;85.02941132;163.0617065;119.0348282; | TXTGC,HH,FF | Saccharides |
| 117 | 44.6 | NEG | 181.0715 | 181.071738 | 1.2 | C6H14O6 | Allitol | 181.0716858;89.02428436;59.01376724;101.0242691;71.01376343;73.02940369;85.02941132;163.0617065;119.0348282; | TXTGC,HH,FF | Saccharides |
| 118 | 116.7 | NEG | 181.0716 | 181.071738 | 0.5 | C_6_H_14_O_6_ | D-Sorbitol | 59.0136528;181.0717926;101.0243759;89.02471161;166.0276031;71.01409912;61.98835754;181.052002;120.9883881; | TXTGC,HH,FF | Saccharides |
| 119 | 373.5 | NEG | 285.04 | 285.040438 | 1.4 | C_15_H_10_O_6_ | Kaempferol | 285.0400085;284.1582336;59.01376343;71.01373291;241.1443329;240.1685791;151.0035706;267.1235962;73.02928925; | TXTGC，RX,MY,DG,HH,GC | Flavonoids |
| 120 | 349.5 | NEG | 301.0352 | 301.035353 | 0.7 | C_15_H_10_O_7_ | Quercetin | 301.0348816;151.0034637;178.9983215;121.0293503;107.01371;273.039978;59.01378632;300.190033;152.0069122;136.0165405; | TXTGC，RX,MY,HJ,HH,WLX,GC | Flavonoids |
| 121 | 498.7 | NEG | 471.3476 | 471.3479602 | 0.8 | C_30_H_48_O_4_ | Lucidumol A | 471.3471985;470.3348389;52.37240982;427.3597717;411.3256226;428.3640442;52.48389435;59.01381302; | WLX | Triterpenoids |
| 122 | 309.6 | POS | 595.1661 | 595.1657703 | 0.5 | C_27_H_30_O1_5_ | Lonicerin | 287.0552979;85.02848053;288.0587158;71.04920959;129.0548401;594.3434448;70.06533813;433.1138;301.0704651; | WLX | Flavonoids |
| 123 | 321.1 | NEG | 193.0506 | 193.050609 | 0.2 | C_10_H_10_O_4_ | Isoferulic acid | 134.0371094;178.026947;193.050415;149.0606079;121.0293503;121.0656357;149.0963135;135.0407104;108.0452728; | WLX | Phenylpropanoids (C6-C3) |

Notes：HTP：Z.ailanthoides Bark, TXTGC：Clematis intricata Bunge, RX：Frankincense, MY：Myrrh, DG：Angelica sinensis, HJ：Sichuan Pepper, CX：Chuanxiong, HH：Safflower, WLX：Clematis chinensis Osbeck, BZ：Dahurian Angelica, GC：Licorice Root, FF：Fang Feng.All substances included in this table were confirmed by matching with authentic standards in the reference library.

**SECTION 3**

**Detailed procedural steps of Manipulation therapy**


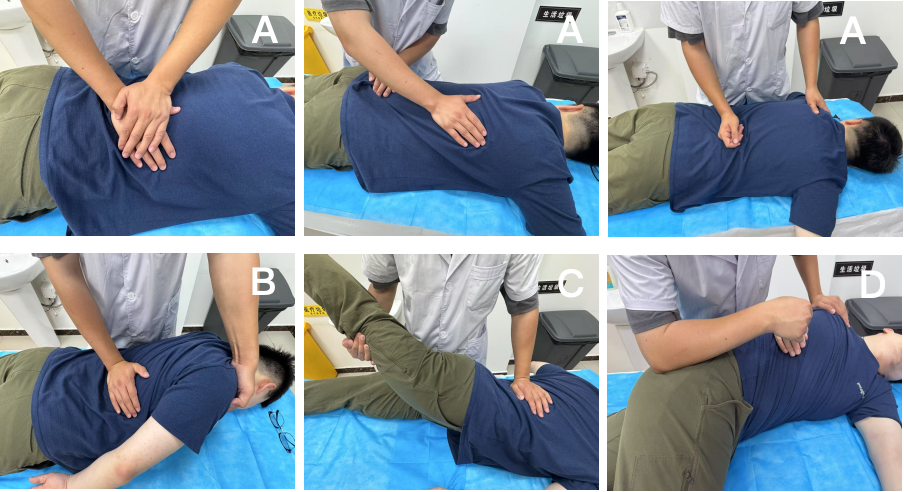


**Supplementary Figure 1**. (A) Muscle-Relaxing; (B) Shoulder Traction with Back Push; (C) Leg Traction with Waist Push; (D) Shoulder Traction with Hip Push.

(A) Muscle relaxation: Patients were placed in the prone position and instructed to fully relax. The practitioner stood on one side and began with a massage along both sides of the spine, following the bladder meridian, using both thumbs or the palm in a downward motion. Upon reaching the Chengfu (BL36) point, the technique transitioned to kneading, proceeding downward through the Gumen (BL37), Weizhong (BL40), and Chengshan (BL57). This was followed by a pressing–pushing technique: the practitioner crossed both hands, with the right hand on top and the left underneath, and applied palm pressure along the spine from the thoracic to the sacral region. Finally, rolling manipulation was performed from the back and waist down to the gluteal and posterior thigh areas, with special emphasis on the lumbar region to relieve muscle spasms and regulate soft tissue tension.

(B) Shoulder traction with back push (搬肩推背): The practitioner lifted the patient’s shoulder with the right hand while simultaneously applying gentle rhythmic pushing–pulling forces to the lower back with the left hand in a bow-like motion. This maneuver was repeated three times.

(C) Leg traction with waist push (搬腿推腰): The practitioner elevated the patient’s thigh with the left hand while applying gentle pushing–pulling forces to the affected lumbar region with the right hand. This technique was also repeated three times.

(D) Shoulder traction with hip push (搬肩推臀): With the patient positioned in lateral decubitus, the upper leg was flexed at the hip and knee in a relaxed manner while the lower leg remained extended. The practitioner pulled the upper shoulder backward with one hand and pushed the pelvis forward with the other. Following several gentle traction movements, the patient was instructed to remain relaxed as the practitioner gradually increased the range of motion. Once joint fixation was perceived, a sudden controlled thrust was delivered. An audible sound from the lumbar region often accompanied this maneuver, indicating successful adjustment.

**SECTION 4**

**Detailed procedural steps of sEMG:**


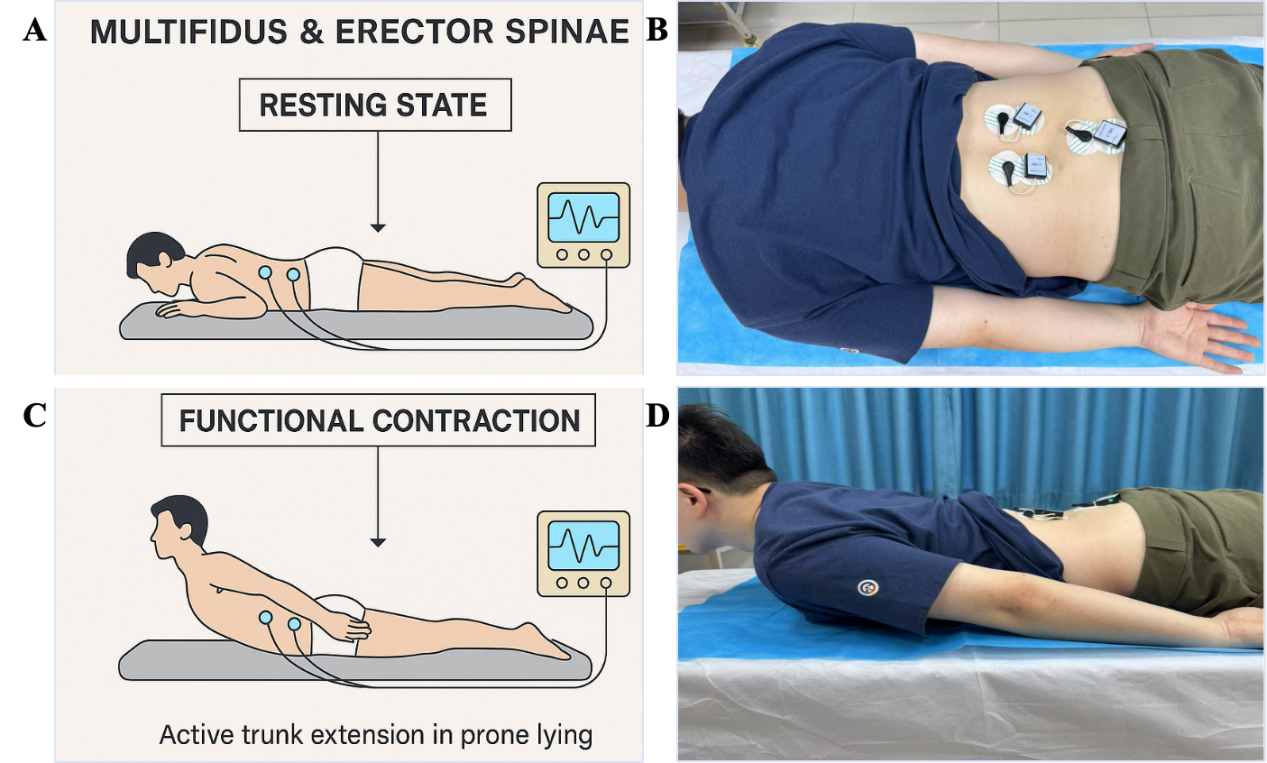


**Supplementary Figure 2**. Schematic of sEMG acquisition. (A, B) Relaxed resting state: the subject lies in the prone position with both hands naturally placed and the body relaxed. (C, D) Functional activation state: the subject’s lower body is fixed, and the upper body is lifted to the maximal functional position ( about 15°) and maintained for 10–15 seconds.

Participants were placed prone with the head turned to one side and arms relaxed alongside the body. After skin preparation to reduce impedance, Ag/AgCl surface electrodes were placed 2 cm lateral to the lumbar 5 (L5) spinous process and 3 cm lateral to the L3 spinous process, aligned along the direction of muscle fibers, with 20 mm spacing between electrodes. A reference electrode was placed over the sacrum. Signals were amplified (gain = 1000), sampled at 2000 Hz, and band-pass filtered between 20 and 450 Hz. During the functional state, participants performed active dorsal extension by slowly lifting the upper body approximately 10–15 cm from the prone position, holding for 5 seconds, then lowering back down, repeating three times. The acquisition procedure is illustrated in Figure 4. RMS and MF values were calculated separately, and all data were averaged for analysis. To minimize inter-individual variability, RMS values were normalized to the percentage of maximal voluntary contraction (%MVC), and MF was calculated from the FFT of the power spectrum to determine the frequency that divides the total energy into two equal parts.
